# Vitalism, Holism, and Metaphorical Dynamics of Hans Spemann’s “Organizer” in the Interwar Period

**DOI:** 10.1007/s10739-022-09682-9

**Published:** 2022-08-19

**Authors:** Christina Brandt

**Affiliations:** Ernst-Haeckel-Haus, Institute for Zoology and Evolutionary Research, Friedrich Schiller University, Jena, Germany

**Keywords:** Holism and vitalism in early twentieth century, History of biology in Germany, Hans Spemann, Metaphors

## Abstract

This paper aims to provide a fresh historical perspective on the debates on vitalism and holism in Germany by analyzing the work of the zoologist Hans Spemann (1869–1941) in the interwar period. Following up previous historical studies, it takes the controversial question about Spemann’s affinity to vitalistic approaches as a starting point. The focus is on Spemann’s holistic research style, and on the shifting meanings of Spemann’s concept of an *organizer*. It is argued that the *organizer* concept unfolded multiple layers of meanings (biological, philosophical, and popular) during the 1920s and early 1930s. A detailed analysis of the metaphorical dynamics in Spemann’s writings sheds light on the subtle vitalistic connotations of his experimental work. How Spemann’s work was received by contemporary scientists and philosophers is analyzed briefly, and Spemann’s holism is explored in the broader historical context of the various issues about reductionism and holism and related methodological questions that were so prominently discussed not only in Germany in the 1920s.

## Hans Spemann’s Holism Revisited

After World War I, a widespread feeling of crisis, as well as a variety of intellectual movements against determinism and mechanistic thinking in a broad sense, dominated cultural discourses in Germany. After the classical Forman thesis (1971), scholars widely accepted the view that within an intellectual milieu hostile to causal analysis and a technical rationality in Weimar Germany, different philosophical approaches flourished that put the very concept of “life” at the foreground.^1^ In the field of science, a parallel (and partly overlapping) broad anti-deterministic movement in debates about the epistemic status of biology itself can be identified. Approaches that tried to distance biological research from a narrow orientation based on a physical or mechanistic paradigm, or that searched for an autonomous theoretical underpinning of biological phenomena, were widespread and flourished in Germany in the interwar period, opening the path to new approaches of organicism (Baedke 2019).

There were several attempts to establish epistemic autonomy of the life sciences. The spectrum of emerging approaches ranged from neovitalistic and holistic perspectives to the search for a theoretical biology. The classical studies of Mitchell Ash (1995) and Anne Harrington (1999) have shown that after World War I, a variety of holistic approaches flourished, especially in psychology, medicine, embryology, neurology, and zoology. Jonathan Harwood, moreover, has criticized the widespread view in the history of biology that “tend[s] to dismiss holists and vitalists as misguided losers in the rise of modern biology” and the often related and extremely simplified historical picture of “Weimar biology” as an “abundance of holists, vitalists and other marginal figures with rather bizarre proposals for the reform of biology, all of them spawned by the peculiar culture which gave birth to National Socialism.” In contrast to a view that assimilates holism and vitalism per se into “reactionary anti-modernist cultural movements,” Harwood argued that the “critics of mechanism endorsed a *diversity* of ontological and epistemological positions” and that these approaches were found “all across the political spectrum” (Harwood 1996, pp. 365–366).

Following Harwood’s argument, this article addresses the historical variety of holistic and vitalistic approaches and questions about modernity in the life sciences in the 1920s and 1930s. It focuses on the work of the zoologist Hans Spemann (1869–1941) as an eminent case of a new experimental *and* holistic approach in early twentieth-century life sciences. Spemann’s research in experimental embryology, particularly his research on the so-called “organizer,” unfolded highly dynamic experimental and theoretical developments in a variety of research fields, thereby fostering materialist as well as vitalist positions in the debates of the 1920s and 1930s.

There have been ongoing discussions in the history of biology regarding whether Spemann should be considered a vitalist. However, the meaning of *vitalism*, which gained new momentum around 1900 after the work of the zoologist-turned-philosopher Hans Driesch (1867–1941), requires more comprehensive historical analysis. In 1969, Viktor Hamburger, a former student of Spemann’s, addressed Spemann’s “psychological vitalism” (Hamburger 1969, p. 1125). Following the English translation of parts of Spemann’s autobiography *Forschung und Leben* [Research and Life] (Spemann 1943), this question re-emerged in the late 1990s (Allen and Maienschein 1999; Hamburger 1999). Garland Allen discussed Spemann’s holism in its broader historical and philosophical context (Allen 2005). Allen described Spemann as a materialist and “ardent experimentalist” who took a holistic or organicist approach, but who was nonetheless neither a vitalist nor a scientist who postulated a “‘vital force,’ supra-organismic directive agency or teleological processes” (Allen 2005, pp. 277–278). Reinhard Mocek advocated the opposite perspective. Depicting Hans Driesch’s vitalism as an early attempt at a systems theory approach in biology that sought to explain self-regulation, Mocek regarded Spemann’s work as leading to a paradigm shift in Germany in the decades that followed. This shift, he argued, began with Driesch’s embryological work in the late nineteenth century and resulted in the abandonment in Germany of classical *Entwicklungsmechanik* [developmental mechanics], with its strictly materialist and mechanistic program (Mocek 1998, pp. 333–342, 384–387).

This article analyses Spemann’s affinity for vitalist approaches. It seeks to offer a fresh perspective by discussing Spemann’s organismic approach within the context of the broader scientific and cultural discourses of the 1920s and 1930s. An analysis of Spemann’s work provides an excellent case to revisit the historical complexity of the then-ongoing mechanist–vitalist debates. In contrast to a subtle but still widespread view that tends to equate vitalistic and holistic approaches with a kind of anti-modernism, one needs to differentiate between a historiographic appreciation of modernity and the (self-)understanding of the historical actors concerning processes of modernization as well as the complex meanings of the notion of modernity more generally in cultural discourses in the Weimar period. By considering how contemporary biologists and philosophers received Spemann’s work, I argue that Spemann was often regarded, at least in Germany, as a representative of a *modern* way of doing biological research precisely *because* of his anti-reductionist, anti-mechanistic program. Moreover, Spemann’s organicist research style had clear vitalist overtones. This, however, did not contradict his strictly experimental program because his daily practical work involved the handling and manipulation of the *living material* of embryos. In one sense, then, Spemann might well be called a materialist because he searched for causal relations in embryonic development. However, he was also someone who rejected any mechanistic view of the living being.

This article has two parts. The first part seeks to understand the specificity of Spemann’s holism by focusing on Spemann’s research style. He combined a modern experimentalist approach with a self-understanding that drew on an older handicraft tradition. Connected to this is the crucial role that analogies between science and the arts and metaphors such as “nature acting as an artist” played in Spemann’s approach. The object under study—the embryo—was seen as having intrinsic agency. The status of the so-called “psychic analogies” (Horder and Weindling 1986, p. 217) for the living material that can be found in Spemann’s work (often cited as evidence of a vitalist approach) are explored in this context. The second part discusses the holistic aspects of the main concept in Spemann’s research, namely that of the *organizer*. Spemann and Hilde Mangold (1898–1924) introduced this concept in the early 1920s to explain the axial system's embryonic development and the neural tube's induction. It became a cutting-edge idea in experimental embryology from the mid-1920s to the mid-1930s. The article traces the different layers of meanings in the concept and argues that the epistemic success of the *organizer* was deeply rooted in its ambiguous and metaphorical character. The article traces different lines of holism and anti-reductionism that were so widely discussed in the 1920s and early 1930s by briefly exploring these metaphorical dynamics in the different phases of Spemann’s research, that of his group, and in the wider research environment of the time.

## Spemann’s Holistic Research Style: Experiments, Observation, and Psychic Analogies for the Living Being

As previously mentioned, Spemann is often regarded as an “ardent experimentalist” and a leading figure in a shift from the evolutionarily framed “descriptive embryology” (in the style of Ernst Haeckel) to “experimental embryology” of the late nineteenth and early twentieth century, which focused on investigating causal and material aspects of morphology and development (Allen 2005, p. 277). However, in the emerging shifts in embryological studies around 1900, historians of science have argued, the central dichotomy was not between observation and experiment but rather an emerging interest in different types of causation (Allen 2007; Nyhart 1995). Whereas for a researcher like Haeckel (1834–1919), causation was primarily *historical* causation and phylogeny ultimately the cause of ontogeny, a younger generation of anatomists and zoologists like Wilhelm Roux (1850–1924) or Hans Driesch (1867–1941), both former students of Haeckel, began to study *mechanical* causation in morphology and development in the 1880s.

Visual representations and their standardization, however, continued to be important elements of embryology during the twentieth century (Hopwood 2005). By showing that descriptive and comparative work on embryos persisted long into the twentieth century, Nick Hopwood has argued that “treating twentieth-century embryology exclusively as a branch of experimental biology is particularly problematic” (Hopwood 2009, p. 286). Instead, Hopwood paints a picture of a crisis in evolutionary morphology and the emergence of a variety of approaches from 1880 up to 1930, a period in which there was not a unified disciplinary understanding of “embryology.” Embryological work was conducted in physiological, medical, anatomical, or zoological institutes, leaving much leeway for a “range of embryologies” and a “wide spectrum of scientific, technical, and medical activities” (Hopwood 2009, p. 286).^2^ Indeed, the very notion of experimental embryology as opposed to descriptive embryology arose from this academic “turmoil” in the 1880s when Roux and others tried to establish a new branch of science called *Entwicklungsmechanik* [developmental mechanics] (Hopwood 2009, p. 298). This approach distanced itself from speculative approaches such as that of Haeckel and instead sought causal explanations whose hypothetical assumptions could be tested experimentally. Roux’s famous experiment on frogs in the late 1880s showed that destruction of one of the blastomeres in the 2-cell stage in early development resulted in only a half-embryo (Roux 1888). Roux’s results, and also August Weismann’s (1834–1914) theory of heredity (Weismann 1885, 1892), supported the mechanistic view that became known as the “mosaic theory,” which claimed that development could be explained in terms of a mechanistic parceling-out of hereditary units during cleavage. Development, according to the mosaic theory, was a process in which the cell’s capacities became increasingly restricted and determined.

In 1891, Hans Driesch, who had also initially worked within a mechanistic framework, obtained contradictory results from his experiments on sea urchins. By separating the two blastomeres (through shaking, not destroying), Driesch did not obtain half-embryos (like Roux) but rather two normal (although smaller) embryos that developed from each blastomere (Driesch 1891). The sea urchin embryo’s ability to overcome the experimentally produced interventions and to develop into a normal “whole” led Driesch to emphasize the fundamental self-regulative power of living objects. Such a “harmonious equipotential system” of living organisms, Driesch believed, could not be explained as the action of a machine-like entity (Driesch 1902). In the early twentieth century, Driesch abandoned experimental research and turned to philosophy, where he further developed his widely received but also highly controversial views on neo-vitalism (Driesch 1905). Allen has argued that “the rise of experimental embryology occurred in two phases: (1) the analytical, reductionist program of Roux’s *Entwicklungsmechanik*, and (2) the holistic yet still causal-analytical work of the Spemann school at Freiburg” (Allen 2007, p. 138).^3^ However, the question remains whether Spemann’s work represents a continuation of a program of experimental embryology that was ultimately rooted in Roux’s causal-analytical and mechanistic approaches or whether Spemann’s holism represented a fundamental paradigm shift.

In contrast to Driesch and other holistic or vitalistic theoreticians such as Jakob von Uexküll (the other famous and influential contemporary sceptic concerning mechanistic approaches), Spemann never explicitly advocated a theoretical standpoint on holism or vitalism. All three figures—Spemann, Driesch, and Uexküll—were born in the 1860s and belonged to the same generation. Spemann did, in fact, criticize Driesch’s idea of “entelechy” on several occasions. However, to infer from this that Spemann distanced himself from Driesch would be only to tell half of the story, because throughout his entire scientific career, Spemann set Driesch’s description of the organism as a “harmonious-equipotential system” as a central pillar of his research. In the main chapter on “induction and the problem of wholeness” of his 1936 book *Experimentelle Beiträge zu einer Theorie der Entwicklung* [Experimental Contributions to a Theory of Development], Spemann discussed the “harmonious-equipotential system” as a serious and prominent question in research, although he distanced himself from Driesch’s idealistic idea of an immaterial *Entelechie* [entelechy].^4^ In 1923, he described the discovery of the “organizer” as providing the unique possibility of “opening up a breach” experimentally in Driesch’s more or less speculative ideas.^5^ However, as Spemann’s early criticism of the very notion of *Entwicklungsmechanik* [developmental mechanics] and his preference for the term *Entwicklungsphysiologie* [developmental physiology] in 1907 indicates,^6^ he was indeed a passionate experimenter who searched for causal factors in development but who rejected with equal passion any kind of reductionistic approach and narrow mechanistic explanation. There are two aspects to be emphasized here: first, Spemann’s conviction that early twentieth century biology had overcome older reductionist theories on development and heredity as advocated by Roux and Weismann in the 1880s and 1890s;^7^ and, second (and related to Spemann’s research style itself), the way in which he described his approach to scientific practice, namely, by referring to traditional ideas of craftmanship.

Spemann clearly saw himself as an experimenter, although not primarily in the modern, more technical or analytical sense. Instead, he seemed to have regarded himself as a skilled craftsman or artisan of the old school in which particularly manual as well as visual skills were required. A talk Spemann gave at the *Volkshochschule* (an adult education program that Spemann had co-founded in Freiburg) about “The Researcher” is very illuminating in this respect. This talk was part of a 1932 lecture series devoted to “Man in Service of Society.”^8^ After raising the question of the social utility of the sciences, Spemann argued vehemently that science as pure research would serve society in the same way as humanities and culture do. Spemann differentiated four types of scientists: The “collector” (epitomized by Darwin), the “taxonomist” (epitomized by Linnaeus), the “experimenter or technician” (epitomized by Leonardo da Vinci), and the “observer” (epitomized by Goethe). While the “observer” aims to “watch … the things in their eternal relations,”^9^ the experimenter had the urge to exercise power. Spemann here resorted to dramatic terms in emphasizing that “it would be indeed a colossal pleasure if one could force a living object to do something that it would not have done without the will of the experimenter.”^10^ The passages in the lecture in which he contrasted the observer and the experimenter and described the experimenter’s motivation in doing science are very illuminating regarding Spemann’s own research approach. He emphasized that the experimenter —besides his urge to power—experiences all things as a “vital flow of powerful drives [*Triebe*].”^11^ The experimenter not only aims to understand the internal correlations of the natural order but, because he wants to become a part of these correlations, aims to put *his own person* into this flow of correlations as the co-creator of the dynamic processes.^12^ Spemann probably would have seen himself in the hybrid figure of an “experimenter,” as so described, and as an “observer,” described in the Goethean sense. On other occasions, Spemann liked to compare his biological laboratory to an old handicraft *Werkstatt* [workshop]. Viktor Hamburger, who completed his PhD under Spemann in the early 1920s, provided a lively retrospective account of the manual skills required in Spemann´s laboratory:

Lecture courses took only a small part of our time. Most of our waking hours were spent at our workbenches and devoted to our experiments. Long before the breeding season of the amphibians began early in April, we were at work, preparing the delicate glass instruments that Spemann had invented for doing microsurgery on the tiny embryos. The operations were performed with glass needles, drawn out to an almost microscopic point in the flame of a micro-gasburner that had been fashioned by extending a tube to capillary size. With these glass needles we cut out tiny fragments of the soft embryo. Steady hands were required, because we worked under a binocular low-power microscope that also magnified our movements. The fragment was then transferred to another embryo in a micropipette and implanted at a specified site. The embryos were moved and turned over with a “hairloop,” another clever invention of Spemann’s: a soft baby’s hair (originally taken from his own offspring) was looped and the two ends were fitted into the capillary opening of a handle that consisted of a piece of glass tubing, then sealed in with hot wax. (Hamburger 1984, p. 9)

Similarly, in a speech at Spemann’s sixtieth birthday celebration (1929), a colleague emphasized the analogy between the Freiburg laboratory and a “*Meisterwerkstatt* [master workshop] of former centuries” as the main characteristic of Spemann’s school. He used the handicraft tradition, particularly the analogy to art schools of famous painters of the past, to describe the atmosphere at Spemann’s institute.^13^ Eckhard Rotmann, another student of Spemann, also stressed in 1943 that his teacher liked to compare his laboratory with a *Werkstatt* [workshop] from former times: “Scientific education and ‘manual’ training were transferred in personal dialogue from man to man, from hand to hand. He was the ‘active master’ whom the others wanted to emulate” (Rotmann 1943, p. 260).^14^

It seemed to be quite common for Spemann’s students to address him as *Meister* [master]. This was not merely rhetorical but shows that the comparison to the handicraft and arts traditions was a central part of the group’s scientific self-understanding. Another point is related to this equation of experimental embryological work with manual and artisan skills, namely, the centrality of *Anschaulichkeit* [vividness or clarity]*.* The manual microsurgery skills needed in the transplantation experiments were the central backbone of laboratory work. Such skills were accompanied by another central aspect, the visual quality of the observation method. What Allen has described as a central characteristic of Hamburger’s research style, namely, “observation and its visual representation through artistic rendering were crucial for doing good biology” (Allen 2004, p. 429), can also be applied to his teacher Spemann. Indeed, in his biographical notes on Spemann, Hamburger himself emphasized the deeply visual quality of Spemann’s approach. He pointed out that “abstract theoretical considerations” had little impact on Spemann; instead, “his original ideas for an experiment were often inspired by a concrete visual experience” (Hamburger 1988, p. 15).

Whereas several painstaking drawings can be found in Spemann’s laboratory notebooks in the early phases of his career, in later phases, and especially after World War I, photographic representations of the developing newts became prevalent in his records. As the archival material shows, Spemann documented each of his microsurgical experimentation series in his experimental years by using specifically numbered notebooks.^15^ Each contained countless, detailed descriptions (handwritten in shorthand) of the *tissue movements* of the developing embryo.

Jane Oppenheimer and others have argued that Spemann was not interested in analyzing cells and “did not construe his organizer … in terms of the sum-of-its-parts-the cells” but rather in terms of a “supracellular agency” (Oppenheimer 1970, p. 77). Spemann’s observational language in his records illustrates supracellular agency. In his practical work, Spemann did indeed first address tissue movements and his observations described the embryo’s plasticity in terms of patterns of regions, dynamic determinations. He also used German terms such as *Gestaltungsbewegungen* or *Gestaltungstendenzen* [movements of forming, tendency to formation] (see, for example, Spemann 1936, pp. 69, 132, 225). The specific shapes of the developing embryo were documented in numerous photos of each transplantation experiment. These photographic representations were a further backbone of Spemann’s observational approach.

With this emphasis on visually representing the research object, his work was, of course, deeply rooted in the nineteenth century embryological tradition. With the media developments that took place after World War I—characterized by the proliferation of visual media in everyday culture^16^—Spemann’s approach gained new momentum. In 1920, Spemann, the artist Ferdinand Leiber, and the zoologist Horst Wachs (a former student of Spemann’s from the University of Rostock) announced the foundation of a *G.m.b.H Bildarchiv Freiburg* (archive for scientific pictures) (Spemann 1920). This *Bildarchiv* had a twofold aim: on the one hand, they wanted to use it to establish a central and publicly accessible collection of diapositives from natural objects or scientific work as well as of drawings by scientists from diverse research fields. On the other hand, this collection opened up new publication possibilities that were faster and also cheaper than traditional journals, which was most important during the precarious economic situation after World War I.^17^ When announcing this venture in 1920, Spemann highlighted the value of modern photographs for research. He described the immediacy, directness, as well as the liveliness of the new medium as its most important advantages. Photographs represented the natural object directly and—even more importantly—individually. They thus became invaluable for experimental research as well as for teaching (Spemann 1920, p. 304).

As becomes clear from Spemann’s article, through the photograph the object under study could speak directly to the observer*.*^18^ This idea of immediacy—not just of observed facts but of the research object itself—was an important aspect of Spemann’s approach and was also characteristic of his own relationship to his research objects, which he sometimes described as an intimate dialogue. A closer look at Spemann’s *Rektoratsrede* [Rectoral Address], *Zur Theorie der tierischen Entwicklung* [On the Theory of Animal Development], is very interesting in this respect. This address was given to an academic audience at Freiburg upon the occasion of his inauguration as rector of the university in 1923. Hilde Mangold’s pioneering experiments had just been completed, and their co-authored article had been submitted for publication. The speech is among the first public occasions where Spemann used the term *organizer* (instead of *center of organization*, a term he had introduced in 1919).

Both the idea of immediacy and the metaphor of “nature as acting as an artist” played important roles in this talk. Spemann used the visual arts metaphor to describe the power of the newly discovered “organizer.” He explained Driesch’s idea of the living organism as a “harmonious-equipotential system” in a teleological way and argued that this principle of life (namely, that it was able to overcome disturbances in the developmental process because it strives toward an “ultimate goal” [*Endziel*]) was already familiar because it was reminiscent of our own human faculties. “Nature,” he explained “proceeds in development in the same way as an artist when he produces a painting or sculpture, indeed in the same way as every organizer proceeds in handling a given material, be it living or nonliving.”^19^

Spemann used this clearly teleological statement to distance himself from his predecessor in the chair of zoology at Freiburg, August Weismann, whose theories were described as mechanistic and deterministic. Against a “Weismannian” theory, where development was seen as running like a “wound-up clock” [*aufgezogenes Uhrwerk*] and guided by an inherited structure, Spemann argued that his discovery of the “organizer” would clearly demonstrate how much the “becoming of the organism” [*das Werden des Organismus*] resembles processes well-known to us from our own activities. Spemann even went a step further by concluding his lecture with a statement about his intimate relationship to the subject under study:

If I may leave the ordinary, dispassionate sobriety of the researcher for a moment on an occasion such as ours today, I must confess that I often have the impression of having a dialogue with my experimental work, in which my conversational partner seemed to be the cleverer.^20^

The “impression of having a dialogue” with the living object and the emphasis on the immediacy of the research object, which, in Spemann’s hands, was (not only metaphorically) seen as a protagonist or conversation partner, were framed by Spemann’s conviction that a good model for biology—that is, for understanding the living world—was not to be found in physics or chemistry but rather in psychology. On the very last page of his book *Experimentelle Beiträge zu einer Theorie der Entwicklung* [Experimental Contributions to a Theory of Development], Spemann reflected on his own metaphors by arguing:

Again and again, terms have been used which designate psychological and not physical analogies. These were not meant to be just poetic analogies. I meant to say that the reaction of a pluripotential embryonic part, when located in a particular ‘situation,’ an embryonic ‘field,’ is not an ordinary simple or complex reaction. It means that these developmental processes resemble, in the manner of their connectivity, most closely those vital processes of which we have the most intimate knowledge: the psychic processes. It means that – quite apart from all philosophical considerations and merely for the sake of progress in our empirical knowledge, we should not miss the chance given us by our position between the two worlds. This insight is now dawning to a few scattered people. I hope that my experiments have made a few steps toward this high goal. (Spemann 1936, p. 278; English translation from Hamburger 1999, p. 243)

The passages in this book and Spemann’s so-called “psychic” analogy, which at first glance seems to contradict his experimental and empirical attitude, have already been discussed intensively by historians of science (Horder and Weindling 1986, pp. 217–219). They are usually interpreted as indicating the influence of August Pauly (1850–1914), who propagated a neo-Lamarckian, psychobiological program as a critique of mechanistic interpretations in Darwinian evolutionary theories (Pauly 1905). He was Spemann’s close friend and mentor when Spemann was in his twenties (Rinard 1988; Allen and Maienschein 1999; Hamburger 1999). In his autobiography, which Spemann wrote in the very last years of his life (in his early seventies), he not only emphasized how important Pauly’s influence had been on him in his younger years but he also underscored the point that, as an old man, he was “more than ever” convinced that the organism was “*beseelt*” in all its living parts (Spemann 1943, p. 167).^21^

To trace Spemann’s psychic analogies back to the influence of August Pauly in the first decade of the twentieth century is certainly important. Horder and Weindling further emphasized that, around 1900, the conception of a psychic dimension to matter and living systems seemed reasonable to many scientists in Germany. They ascribe this to Haeckel’s overwhelming “tradition of monism, unifying the physical with the psychic,” Richard Semon’s 1904 mneme theory, and other related developments at that time (Horder and Weindling 1986, pp. 217–218).

However, to describe Spemann’s use of psychic analogies only as a kind of outmoded and obsolete aftermath of an earlier influence of neo-Lamarckian positions around 1900 captures only a part of the historical complexity of the debates in the 1920s and 1930s. At this time, fundamental critics of the late nineteenth century *Maschinentheorie* [machine theory] of organisms also laid the foundation for new perspectives of clearly anti-vitalist theoreticians such as Julius Schaxel (1887–1943) or Ludwig von Bertalanffy (1901–1972).^22^ Moreover, a variety of approaches flourished that aimed to understand the specificity of organismic processes. Especially in vitalist positions of the 1920s, references to the psychic seemed to have increased. In 1929, the philosopher Adolf Meyer-Abich (1893–1971) emphasized that “psychobiologism” was a main feature of all kinds of Aristotelianism in biology (Meyer 1929, p. 11), and he saw prominent vitalist positions, such as those of Driesch and Uexküll, as neo-Aristotelian approaches. Indeed, both used terms with reference to the psychic. Driesch replaced the term *entelechy* with the phrase *psychoid*. Uexküll also used the term *psychoid* to describe the specific relationship between an organism and its environment. For Meyer-Abich, Uexküll’s terminology for progressively describing vital phenomena such as *mechanisator* (the immaterial factor that controls bodily mechanisms), *psychoid* (the specific connectivity of organism and environment), and *psyche* was in principle a new articulation of Aristotle’s theory of the soul (Meyer 1929, pp. 13–22). In addition, in the German philosophical debates at least as far back as the work in the 1880s of Wilhelm Dilthey (1833–1911), the epistemic status of the newly developing discipline of psychology in its relation to physiology had itself been at the center of very lively discussions about the demarcation between the natural sciences and the *Geisteswissenschaften* [humanities], as well as related questions about their methodological differences. In his contribution to the *Festschrift* for Spemann’s sixtieth birthday, Uexküll described biology as being situated precisely between physiology and psychology (Uexküll 1929). In the context of other holistic approaches, diverse new attempts at an animal psychology [*Tierpsychologie*] emerged in physiology.^23^ Furthermore, a variety of ethological approaches developed in zoology in the early twentieth century, largely independently of theoretical vitalist approaches. These also aimed at a non-reductionist (that is, non-behavioristic) understanding of animal behavior. Spemann was in contact with one of the leading younger scientists in that field, namely Karl von Frisch (1886–1982).

Thus, a rejection of physicochemical reductionism and mechanistic approaches, as well as the theoretical search for a new epistemological foundation for the science of life—even beyond the vitalist-mechanist debates—was one of the main discourses in German biology at that time. Spemann’s use of psychic analogies, besides their clear vitalist orientation, must also be understood in these broader contexts, namely, as a tentative search for another kind of epistemic orientation for biology, distinct from physics or chemistry, and one that emphasizes the autonomy of the living organism.

It was precisely in this way that some contemporary scientists received the last section of Spemann’s book. Hans Petersen (1885–1946), a former student of the anatomist Hermann Braus (1868–1924, who had been a friend of Spemann’s from his days in Würzburg) and Braus’s successor as professor of anatomy at the University of Würzburg, wrote a review of Spemann’s book in 1937.^24^ Petersen not only built a bridge between the nineteenth century work of Johannes Müller to Spemann’s results, but he also enthusiastically emphasized that the psychic analogy of “our first and most critical experimental embryologist” could be taken as “proof that biology was about to be free from the shackles of an a priori mechanistic and materialist world of thoughts” and that it was on its way to becoming an “autonomous science of the autonomous life.”^25^ Hence, Spemann was for many of his contemporaries clearly a holist (if not a vitalist), and with his critiques of reductionist mechanistic models in biology, he seemed to be a representative of the main discourse at that time.^26^

The specificities of Spemann’s approach and research style, as described above, were also recognized by his contemporaries—and not just by biologists. In his obituary of Spemann in 1941, the zoologist Alfred Kühn (1885–1968), who had studied with Weismann in Freiburg,^27^ associated Spemann rhetorically with the Goethean approach to nature when he wrote: “for him [Spemann], the experiment was not a compulsion using levers and screws, but rather like a dialogue with a living working partner.”^28^ For Kühn, Spemann embodied the ideal type of scientist. “Not only your achievement,” Kühn wrote to Spemann in 1929, “but above all your whole person represents for me that which one should strive for.”^29^ In a similar vein, Walther Vogt (1888–1941), professor of anatomy in Munich and Zurich, emphasized Spemann’s “intuition, fantasy and sensitivity for the essence of design [*Wesen der Gestaltung*]” in 1929.^30^

For the philosopher Martin Heidegger (1889–1976), Spemann’s way of doing science even provided the ideal model for a dialogue between biology and metaphysics. Heidegger saw in Spemann—in addition to Uexküll and Driesch—one of the main leaders of a new kind of a biological science that tried “to protect itself against the tyranny of physics and chemistry,” a biology that would fight for its own essence (Heidegger [1929/1930] 1983, pp. 277–278).^31^ For the philosopher, this fight was inherently a search for a metaphysical interpretation of life, and the new organicism and holism of that generation of biologists, such as Driesch, Uexküll, and Spemann, was a clear break with the older mechanist and machine-like thinking that Heidegger had criticized. In his lecture course *Grundbegriffe der Metaphysik* [*The Fundamental Concepts of Metaphysics*] from the winter semester of 1929/1930, Heidegger, who had just succeeded his former teacher and the founder of phenomenology, Edmund Husserl, as the chair of philosophy in Freiburg, extensively discussed the notion of “life” from an ontological perspective. Aiming at understanding human existence in its relations and differences to other living beings, Heidegger also raised the problem of the unity of science and metaphysics. For him, a fundamental step toward a metaphysical underpinning of the science of life would lie in the “leadership of a researcher” [*Führerschaft eines Forschers*], which, according to Heidegger, became especially apparent in the “nativeness of an intimate connectivity” of the scientist and his subject under study.^32^ It was within this context that Heidegger referred to Spemann’s “ingenious research” (Heidegger [1929/1930] 1983, pp. 381, 279–280). In his study of Heidegger’s relationship to National Socialism, the historian Victor Farias has pointed out that the philosopher tried to encourage Spemann to contribute to establishing a new *Weltbild* [worldview] in the late 1920s.^33^ On the basis of papers in Spemann’s archive, however, it is not possible to reconstruct whether Spemann and Heidegger engaged in a deeper cooperation. In a letter to his son Ulrich, Spemann declared in the late 1930s that Heidegger’s philosophy remained extraneous to him and that he preferred Husserl’s personality.^34^

The appreciation of Spemann’s work by two very differently oriented theoreticians, namely Ludwig von Bertalanffy and Jakob von Uexküll, is especially illuminating concerning the internal debates in biology in the interwar period. Around 1930, Bertalanffy developed an early approach of a “system theory of life” [*Systemtheorie des Lebens*] and the basic principles of “organismic biology,” as opposed to both vitalism and mechanistic materialism. He referred in detail to Spemann, and he described his work as the second phase of a research program that was ultimately traceable to Driesch. (Bertalanffy, however, clearly delineated Spemann’s idea of wholeness—as a description of a spatial and temporal overall condition of the living system—from Driesch’s idea of wholeness—as a description of a final state or goal, guided by a factor *x* that Driesch had called entelechy (Bertalanffy 1930/1931, pp. 391, 402, 388)). For Bertalanffy, Spemann had clearly solved the main problem of determination, and Spemann’s research on the *organizer* provided one of the important building blocks of Bertalanffy’s theory of the organism. However, the Austrian theoretician also saw the danger that Spemann’s results could be interpreted in a vitalistic manner, especially because Spemann himself would facilitate such an interpretation. Bertalanffy referred explicitly to Spemann’s “classical Rectorate speech,” and, by criticizing Spemann’s use of terms such as “Formbildungsstreben” [*striving* to formation], Bertalanffy drew attention to Spemann’s self-portrayal of having a dialogue with the living object in his experimental work (Bertalanffy 1928, pp. 196–197).

Whereas Bertalanffy distanced himself from vitalistic and idealistic underpinnings of Spemann’s approach, Jakob von Uexküll explicitly appreciated this. The Estonian-born biologist (who introduced the concept of *Umwelt* [environment] to biology) was developing ecological and biosemiotic perspectives on the relation of organism and environment. He built up a new organism-centered theoretical biology on the basis of a reinterpretation of Kantian philosophy during the 1920s.^35^ Although (the politically right-wing) Uexküll was involved in a quite different research field, he recognized early on the future potential of Spemann’s research on the *organizer* for a holistic study of development and heredity. Uexküll even spoke quite enthusiastically about “Spemannism,” which he juxtaposed with “Mendelism.” In the second edition of *Theoretische Biologie* [Theoretical Biology] from 1928, he discussed “Spemannism” and “Mendelism” as the two main facets of the new biological research on the organism that contributed different but complementary perspectives on issues of heredity and development. Whereas for Uexküll “Mendelism” represented a science of heredity in a narrow sense, dealing with the materiality and difference of hereditary factors, “Spemannism” represented a science of the principles of formation. Uexküll used extensive metaphors from the field of music to explain the necessarily complementary character of both research approaches. Whereas “Mendelism” concerned the relatedness of hereditary factors in a narrow sense (analogous to notation theory in music), “Spemannism” concerned a comprehensive science of composition, “like the composition theory in music.”^36^ When Uexküll vehemently rejected pure materialism, however, his metaphors became more drastic:

It is undoubtedly correct that each written note in music is materially an inkblot, hence every characteristic of living beings is undoubtedly something committed to matter. However, to want to perceive nothing more in the characteristics of living beings than the twitching of atoms [*Atomgezappel*] is not only to be hard of hearing [*Schwerhörigkeit*] but in principle to be deaf [*Taubheit*]. In this situation, it is completely hopeless to convince those researchers who see only mechanical and chemical problems that biological problems also still exist.^37^

## The Concept of the *Organizer*—Metaphorical Dynamics in the 1920s

As Tobias Cheung has shown, Jakob von Uexküll referred frequently to Driesch’s and Spemann’s work, and especially to the work on the *organizer* in the 1920s (Cheung 2004, p. 146). Indeed, in the 1920s and early 1930s, research on the *organizer* flourished, not only in Freiburg but also internationally. Scientists from different fields were fascinated by the results emanating from Spemann’s group in Freiburg, and contemporary scientists even spoke of a “gold rush” atmosphere in experimental embryology. In 1935, Spemann received the Nobel Prize in Physiology or Medicine for his discovery of the *organizer* effect in embryonic development.

Spemann’s famous research on the *organizer* flourished in his Freiburg years, but this success was rooted in his technical approaches developed prior to the Freiburg period. Already during World War I, when Spemann was a director of the Kaiser Wilhelm Institute for Biology in Berlin, he conducted transplantation experiments with two (differently pigmented) newt embryos (*Triton taeniatus* and *T. cristatus*) (Spemann 1921). He observed that tissue from a specific region of the embryo, the dorsal blastopore lip,^38^ produced a secondary axial system when it was transplanted into the ventral region of a developing host embryo. After Spemann moved to Freiburg, a PhD student, Hilde Pröscholdt (who later married Otto Mangold, Spemann’s assistant) performed more than 200 heteroplastic transplantations of the blastopore’s dorsal lip into the ventral region of a host gastrula in the years 1921 and 1922. In several significant cases, she observed that a secondary axis with neural system developed in the host embryos.^39^ Moreover, it became clear from her experiments that the secondary axial system was built up by the host (and not only the transplanted) tissue. Spemann and Hilde Mangold therefore concluded that the embryonic region of the dorsal blastopore lip was able to induce embryonic development and called this specific embryonic region an “organizer” (Spemann and Mangold 1924).

In the years that followed, more than two dozen PhD students in Spemann’s group systematically explored a huge variety of research problems concerning the observed *organizer* effects, such as the spatial and temporal aspects and, in the late 1920s, xenoplastic transplantation experiments between *Urodela* and *Anura* (Spemann 1924a, b, 1927a, b, c, 1929; Spemann and Schotte 1932). In the 1930s, Johannes Holtfreter (1901–1991), a former student of Spemann’s who later worked with Otto Mangold (who had, in the meantime, become director at the Kaiser Wilhelm Institute for Biology), showed that even dead (killed by heat) or dried (by alcohol) tissue of the dorsal lip was able to induce the embryonic development of a secondary axial system. This caused a wave of excitement in the international embryological research community. These experimental results also had enormous symbolic value and were the beginning of a race to elucidate the material characteristics of the *organizer.* This stimulated a new wave of biochemical approaches in the international field of embryology in the early 1930s.

In the history of biology, Spemann’s work on the *organizer* and his contribution to twentieth century life sciences has been analyzed intensively in recent decades.^40^ However, it has been noted in a critical vein that the experimental work that provided the empirical basis for this conceptual framing was mainly performed by Hilde Mangold, who died in a tragic accident shortly afterwards. The question of whether Spemann gave her enough credit for her contribution has also been at the center of the discussion (Hamburger 1984; Fäßler 1996). Indeed, the way that Spemann interacted with Hilde Mangold (compared with his behavior towards his male students) accords with the view that female scientists of the time were treated unequally. Furthermore, although the concept of the *organizer* is often described as a key concept in twentieth-century biology, its usefulness and appropriateness has also been hotly debated in biology as well as in the history of science.

For biologists, the apparent vitalist aspect of the concept became problematic, especially after World War II. For some historians, the centrality of the *organizer* in Spemann’s work indicates the presence of an “authoritarian social thought” (Horder and Weindling 1986, p. 220). Horder and Weindling argued that Spemann’s *organizer* bears an analogy to his political attitude: “The ‘organiser’ arose from *Sehnsucht* for order, as a means of national survival” (Horder and Weindling 1986, p. 221). They even suggest that the concept had the semantic undertone of a “virtual dictatorship” in the realm of embryonic development: “The concept of the ‘organiser’ represented a similarly dictatorial explanation in supracellular terms” (Horder and Weindling 1986, p. 220). Fäßler (1997), in contrast, draws a balanced picture of Spemann’s political attitudes: Spemann was clearly a political conservative during the Weimar Republic, and like many national conservatives of his generation, he seems to have partially agreed with the National Socialist political system after 1933. However, he also, in some respects, kept a critical distance from it. Fäßler emphasized Spemann’s sympathy with the “*Bekennende Kirche* [Confessing Church],”^41^ which was clearly a statement against National Socialism, and his rejection of any racist and anti-Semitic ideology. Fäßler also stressed that Spemann used his scientific standing to help Jewish colleagues who were forced to emigrate, although he never publicly criticized National Socialist politics (Fäßler 1997, pp. 86–97). However, I will not here discuss in detail the historical debates on how Spemann’s ambivalent political positions and his scientific thoughts, exemplified by the *organizer* concept, could fit together. Instead, by considering the broader historical and cultural context, I will focus on the development of the semantically fluctuating concept of the *organizer* itself, on its multiple layers of meanings, and on the research dynamics that unfolded because of these ambivalences.

The concept of an *organizer* can be regarded as a kind of *boundary concept* par excellence—in the sense of how historians of science have analyzed the epistemic role of such loose and “traveling” concepts in organizing research fields as well as the role of imprecise concepts [*unscharfe Begriffe*] and metaphors in recent decades.^42^ It was precisely because of its scientific vagueness and its meandering meanings that the concept of the *organizer* could have such rhetorical and scientific power. Experimental research on questions about *organizer* effects became a “hot spot” in very different scientific approaches throughout the 1920s and 1930s. I will discuss three main aspects in more detail. First, Spemann himself not only avoided giving a precise definition of the *organizer* concept but, moreover, he became increasingly metaphorical when he tried to describe what exactly the term *organizer* could mean and how *organizer* effects—beyond the mere empirical observations—could be understood. Thus, while Spemann never tried to integrate the observed facts and empirical results into an all-encompassing theoretical framework, the notion of an *organizer* was part of a network of other metaphors and guiding analogies in Spemann’s work that were central to his approach, as I will discuss below. Second, a historical analysis of the use of the term *organizer* and the metaphorical dynamics related to this term in its wider usages in the 1920s illustrates that the concept had at least three overlapping but different layers of meanings. In addition to its narrow biological use as a metaphorical description of the actions of the dorsal lip in embryonic development, the term had philosophical and political or popular connotations. Third, with respect to its political overtones, the interpretation of the scientific concept in popularized fields seems to have changed, particularly from the 1920s to the 1930 and the rise of the Nazi regime. In the following sections, I will explore these different aspects in greater detail.

## The “Magic Word”: From the “Center of Organization” to the “Organizer”^43^

Spemann never gave a precise definition of the *organizer*, nor did he argue that the concept belonged to the established scientific vocabulary of biology. In 1936 he proclaimed that he had never aimed at creating a “theory of the organizer” [*Organisatortheorie*], but rather that he (still) treated the term as a preliminary, albeit useful, designation (Spemann 1936, p. 275).^44^ In fact, Spemann’s use of the concept changed after it was first introduced. As early as 1919, Spemann had spoken of a “center of organization” [*Organisationszentrum*] before he later (around 1921) used the word *organizer*. In 1919, the problem of biological individuality was the main theoretical framing for the issue—the “primordial problem” [*Urproblem*] of biology, as Spemann put it then. Indeed, the biological questions about individuality were still at stake for many biologists in the 1910s from different perspectives—as, for example, the work of the young Julian Huxley indicates in the context of evolutionary theories (Huxley 1912). At that time, Spemann interpreted the results of his constriction and transplantation experiments on newt embryos (which produced double-headed creatures, among other things) such that the cells of the dorsal lip [*Zellen der oberen Urmundlippe*] were seen as *representing* the individuality of the embryo. Thus, Spemann’s idea of a “center of organization” first emerged in the context of the problem of how the individual organism is determined. Moreover, the term clearly (although not explicitly) pointed toward ideas of an embryonic *Bildung* [formation] of biological individuality (with all the complexity that the term *Bildung* in German implied at that time) (Spemann 1919, pp. 584, 591).^45^

Two years later, in a 1921 postscript (concerning Hilde Mangold’s then-ongoing experiments) added to another article on newt chimeras, Spemann used the word *organizer* [*Organisator*] in a publication for the first time. He introduced the word to refer to a specific part of the region of the organization. The action of the newly discovered “organizer” was described as able to turn “indifferent tissue” into tissue, capable of “self-organization.”^46^ In the same postscript, Spemann emphasized that the effect of the “organizer” was definitely not a “determining influence” but more an “activating influence.” He summarized this view as follows: the “organizing activity” is not an activity of “instruction” but an activity of arrangement.^47^

The first detailed and elaborate discussion of the new concept can be found in an article describing Hilde Mangold’s results Spemann co-authored with Mangold (Spemann and Mangold 1924). In their classic paper, “Über Induktion von Embryonalanlagen durch Implantation artfremder Organisatoren” [On the induction of embryonic primordia by implantation of organizers from different species], Spemann and Mangold first emphasized the provisional nature of the term by arguing that the terms *center of organization* and *organizer* could later prove to be inappropriate in light of future research and be replaced with more detailed notions and clearly defined terms (Spemann and Mangold 1924, p. 636). The idea of an *organizer* was based on the general assumption of a progressively spatial determination of the embryo over the course of temporal development. However, both authors insisted that they had chosen the word *organizer* merely in contrast to the possible idea of a *determiner* [*Determinator*], because the “center of organization” should *not be* regarded as a center that controls the development of the embryo. In this context, they concluded:

The name “organizer” (instead of “determiner”) should express that the effect stemming from these preferred parts [of the embryo, C.B.] is to determining toward a specific, limited direction, but that this effect possesses all the mysterious peculiarities which we know only from living nature.^48^

Spemann and Mangold thus seemed to have intentionally avoided a term such as *determiner*, and it can only be assumed that this was related to Spemann’s rejection of a Weismannian perspective. A notion like *determiner*, with its strong semantic reference to Weismann’s idea of determinants [*Determinanten*], would not have been appropriate to Spemann.^49^ Hence, while the *organizer* was *not* primarily introduced by Spemann and Mangold as a hierarchical concept, or even as a “leadership analogy” (Horder and Weindling 1986, p. 219), in this emphasis on the “mysterious peculiarities” of the living being it becomes clear that, at this time at least, Spemann’s use of *organizer* was certainly embedded within a holistic view that had strong vitalist overtones. Spemann’s former student Hamburger summarized this clearly in 1969 when he wrote:

The organizer concept symbolizes, perhaps subconsciously, Spemann’s deep and strange conviction that all vital phenomena, including, of course, the doings of the embryo, are the emanation of a psychic force akin to, or identical with, the workings of our mind. (Hamburger 1969, p. 1125)

## Multiple Layers of Meanings and Metaphors

The “magic word” *organizer* clearly had “mysterious undertones” (Hamburger 1969, p. 1123). However, to reduce the *organizer* to these vitalist underpinnings would be to see only a small part of the story. As a vague concept, the *organizer* was embedded in a network of other metaphors that rhetorically expressed Spemann’s view of nature. These metaphors are all the more striking because Spemann’s writing style was otherwise very descriptive and extremely modest. In clear contrast to Uexküll or Driesch, for example, Spemann avoided any kind of comprehensive theorizing and, with the exception of the term *organizer*, he was quite reluctant to introduce any kind of new theoretical or speculative terminology. The most prominent of the metaphors that Spemann used in the context of describing what the essence of the *organizer* might be was the psychic analogy found in the last passage of his 1936 book (Spemann 1936, p. 278; see my discussion above).^50^ On some other occasions, Spemann also used the metaphor of gear-wheels (that only loosely interacted as an intentional contrast to the longstanding metaphor of nature as a “clockwork”) in order to demarcate his own approach from the strong mechanistic reductionism that he saw in Weismannism (Spemann 1936, p. 166; 1921, p. 534). Additionally, there are passages in his 1936 book in which Spemann explicitly painted a picture of the *organizer* as an “organizational force” that “shapes the whole” by acting *above* the material, which raises the question of the “*essence* of *Entwicklungsgeschehen*” (Spemann 1936, pp. 94–95).^51^ Finally, another important metaphor in Spemann’s writings was that of “nature acting as an artist,” which Spemann used prominently in his 1923 Rectorate Speech, as discussed above.

These metaphors did not escape the notice of Spemann’s contemporaries, especially in the United States. In 1936, on the occasion of the awarding of the Nobel prize to Spemann, the Princeton zoologist Edwin Grant Conklin (1863–1952), who himself surely had the intention of arguing against “hard boiled” mechanistic approaches and their “absurdly simple explanations” (Conklin 1936, p. 194), emphasized Spemann’s metaphor of nature-acting-as-an-artist sympathetically and at length. In addition to Spemann’s Rectorate Speech, Conklin quoted extensively from a talk that Spemann gave when he visited Woods Hole for several weeks in 1931:

[I]n an address at the Marine Biological Laboratory in Woods Hole in 1931 he [Spemann] closed by saying: “It is my personal conviction that the processes going on in the living matter may be compared with nothing else so well as with the workings of our own mind. To deal with the living organism as if it were animated unto its last fibers seems to me the best way to understand it.” (Conklin 1936, p. 194)

Not every biologist in the United States was as receptive to Spemann’s metaphorical interpretation of the transplantation experiments. Spemann’s friend, the embryologist Ross Granville Harrison (1870–1959), valued Spemann’s empirical discovery but was also cautious about the term *organizer* because the “word may be readily taken to imply more than we are really justified in attributing to the thing itself” (Harrison 1933, p. 317). The embryologist-turned-geneticist Thomas Hunt Morgan (1866–1945) not only criticized the lack of empirical evidence supporting the concept but also rejected any vitalist overtones concerning a supposed “mysterious influence” (Morgan 1927, p. 239). In his *Experimental Embryology* from 1927, Morgan wrote:

But even to assume that because a region develops faster than the rest of the embryo, it becomes therefore the organizing center (here the dorsal lip) is little more than begging the question at issue, because there is no other evidence to appeal to, which proves that more rapid development in one part than in another initiates the kind of change that would make it the organizer of such a structure as the dorsal lip of the blastopore. (Morgan 1927, p. 438)

It is important to note that the vitalist overtones of the biological *organizer* concept surely reflected only some aspects of its manifold meanings. In the way the word *organizer* was used in biological contexts, overlapping connotations came into play from the fields of philosophy (by evoking meanings that, as will be discussed later, connected with specific philosophical traditions) as well as culture and society (where meanings changed between function and instruction). A few sentences on the history of word’s usage might be illuminating to understand this intermingling.

In everyday language, the German word *Organisator* had, first of all, a *functionalist* meaning: An organizer was (is) somebody who is able to structure processes in a good, planned way.^52^ The word itself, while unknown until the early nineteenth century, became more commonly used from the mid-nineteenth century onwards, where it was often used in more narrow and personalized contexts (mainly in historical descriptions of great men as political or military organizers).^53^ However, throughout long periods of the nineteenth century, the word was not used widely in the German language. It was only from late nineteenth century onwards that it found widespread use.^54^ The spread of the word in the twentieth century was attended by a shift in the contexts of its usages, because the organizer was now also used in a more abstract way to refer more generally to somebody who had a specific organizing function within society or a subgroup of society. Thus, the increasing use and shifting meanings of the word *Organisator* around 1900 is another example of a more general cultural shift toward the ideas and practices of functionalism and structuralism in the early twentieth century. This was accompanied by new analytical perspectives on “systems” and on the interaction of their elements—a tendency that can be found in several fields within the arts and the political, social, and natural sciences in the first decades of the twentieth century.

This context is crucial in understanding Spemann’s introduction of the term into biology, and especially the dynamics it unfolded. The very modern idea of functionalism was also contained within the biological notion of an *organizer*. Depending on whether the focus was on an *organizer* as an active agent or on a shortcut for processes of organization, the word conveyed both the seed for a very modern perspective on biological processes in terms of systems and functions and, at the same time, anthropomorphic connotations that referred back to the much older vitalist sense of an organizing agency in the realm of living beings. It was precisely these ambivalences and tensions between different meanings that made the concept so productive on an epistemological level, as these tensions fit perfectly into the debates in biology in the 1920s, where a variety of holistic approaches (a spectrum that ranged from neo-vitalism to holistic materialism) struggled with reductionist views of authority.

Another example from Spemann’s work may be helpful in understanding this ambiguity and, in particular, how very different, even diverging perspectives could become intermingled in his work. When Spemann was working on lens induction, 1903–1907, the notion of a “double assurance” [“*doppelte Sicherung*”] was crucial to understanding inductive processes. As Spemann himself emphasized, he introduced the phrase *double assurance*, which emerged in the engineering field and modern technological domains, after adopting it from his former Würzburg colleague Hermann Braus (Spemann 1936, p. 60). Thus, Spemann assumed a (technical) perspective on the living being precisely at a time when he was still very much inspired by the psycho-Lamarckism of his friend August Pauly and the view of life as analogous to a psychic entity. In an insightful analysis, Rinard (1988) has argued that during those early years, with the example of the “double assurance,” “Spemann combined an increasingly highly prized technical virtuosity with a neo-Lamarckian view of nature” (Rinard 1988, p. 95).

The concept of the *organizer* combined modernity and traces of the past in a similar way. Indeed, the functional aspect of the *organizer* came more and more to the fore in the course of the Spemann group’s research in Freiburg during the 1920s and also in the international reception of their work. The research on the *organizer* was increasingly framed in language devoted to the description of the action of systems, subsystems, and their communication, interaction, and regulation. In particular, the younger generation of Spemann’s students seemed to have turned from Spemann’s style of holism to a thinking that was more along the lines of a modern systems approach. This research led to a further differentiation in terminology, for example, speaking of “organizers of a second order,” where the material interaction of transplanted and host tissue became framed as the interaction of subsystems. Hamburger proposed the term *Aktionssystem* [system of action] for Spemann’s term *induction*, and Otto Mangold spoke about a *Reaktionssystem* [system of reaction] (Bautzmann 1955, p. 288; Spemann 1936, p. 49). Around 1930, the work of Johannes Holtfreter (1901–1992), who was then a member of Otto Mangold’s group at the Kaiser Wilhelm Institute for Biology at Dahlem, demonstrated that even boiled or otherwise dead tissue could produce the effects of the supposed *organizer*. Following this, the focus of research turned more and more toward analyzing the “system of reaction.” For Spemann’s students, then, this shift from the inducing stimulus to the responding system was also accompanied by a shift in the epistemic perspective: “What seemed imposition of organization by an outside agency was really a major part *self-organization* within the reacting system” (Hamburger 1969, p. 1123).

A more materialist reception of the work on the *organizer* proved fruitful in the United Kingdom. Among the best-known of these efforts was those of a Cambridge-based group of scientists centered around Joseph Needham (1900–1995) in the 1930s. Needham tried to elucidate development via biochemical embryology. Erik Peterson has analyzed in detail how Spemann’s concept of an *organizer* became a “catalyst” in the formation of a social network, the “Cambridge Theoretical Biology Club” (Peterson 2014, p. 286), whose members (including Needham, Joseph Henry Woodger [1894–1981], Conrad Hal Waddington [1905–1975], and others, were also inspired by Alfred North Whitehead [1861–1947]) intensely discussed the philosophical issues of a materialist organicism in the mid-1930s (Peterson 2014, 2016). Julian Huxley (1887–1975) and Gavin de Beer (1899–1972) were among those who realized the potential of Spemann’s experimental results quite early on, even before the biochemical turn. Huxley, who in the early 1920s was also in contact with Richard Hertwig in Munich and Richard Goldschmidt at the Kaiser Wilhelm Institute for Biology in Berlin,^55^ contacted Spemann in 1923. In September of that year, Huxley wrote to Spemann asking for a thorough overview of Spemann’s work and complained about the “lack of knowledge” of Spemann’s embryological work in the United Kingdom. Huxley appealed to “do what we can, in this era of unfortunate and exaggerated nationalism, to keep alive the international character of science” and asked Spemann to submit a paper to a newly founded British journal.^56^ He had apparently invited Spemann not only to submit an article, but also to visit the country. In 1924, with the occupation of the Rheinland by the Allies of World War I still ongoing, the political nationalist-conservative Spemann expressed hesitation, although he showed great interest in visiting the British reform schools.^57^ Huxley and Spemann finally met when Spemann gave a lecture tour in the Netherlands in March 1924. At this meeting, Huxley had already surmised a possible relation between Spemann’s work on *organizers* and the ideas of embryonic “gradients” that had emerged from the work of the Chicago zoologist Charles Manning Child (1869–1954).^58^ Gavin de Beer, an embryologist from Oxford, visited Spemann’s group in Freiburg for several weeks in 1926.^59^

Spemann finally gave a lecture tour in London, Cambridge, and Oxford on the occasion of his Croonian Lecture (the Royal Society of London’s premier lecture in the biological sciences), which he delivered in November 1927 (Spemann 1927c).^60^ In Huxley and de Beer’s 1934 comprehensive overview of the state of the art in embryology, *The Elements of Experimental Embryology* (a book they dedicated to Ross Harrison and Hans Spemann), the concept of the *organizer* played a crucial role. At this time, the excitement about biochemical research on the *organizer* was at its peak, and there was also fascination regarding the attitude of manipulation where “[t]he result of grafting an organiser into a suitable environment is just as definitely causally determined and predictable as the result of mixing two known reagents in a test-tube” (Huxley and de Beer [1934] 1963, p. 12). They saw the biochemical attempts as a promising way of reconciling the physiological study of biological order with a physico-chemical analysis. For Huxley and de Beer, the effects of an *organizer* had to be understood as a part of a theory of *gradients*, and in describing the still biologically unclear effects of the *organizer*, their usage of the term necessarily became very metaphorical. The way they depicted the supposed agency of the *organizer* is another example of the capacity of metaphors and loose concepts to evoke new meanings and to inspire new research perspectives when transferred to different research contexts. In 1934, they wrote:

The action of the organiser, then, must be considered as taking place in two phases. First, working as part of the gradient-field, the organiser may be figuratively said to sketch out the presumptive regions in pencil, and then, after invagination, the organiser goes over the same lines with indelible ink. At the same time, the organiser is capable of roughing out the sketch straightway in ink, without any previous pencil work, as in those experiments in which the organiser is grafted into the flank of another embryo. Neural folds can arise from the pencilling alone, and from the inking alone, and this duplicity of methods whereby neural folds can be formed is another example of the principle of “double assurance.” (Huxley and de Beer [1934] 1963, p. 139)

In Germany, especially among biologists of Spemann’s own generation, an idealist interpretation of the *organizer* seemed to be predominant in the 1920s, at least among those scientists who belonged to the holistic or vitalistic camp. Already in 1922, Julius Schaxel (himself clearly not a vitalist but a Marxist and dialectical materialist)^61^ had appreciated that Spemann’s approach would overcome the “inflexibility of a developmental mechanical machine of determination,” but he also warned that Spemann’s research could lead back to former theories of the “idealistic embryologists.”^62^ In his 1928 *Theoretische Biology* [Theoretical Biology], Uexküll proclaimed “Spemannism” to be a new research direction that promised to overturn “Mendelism” (Uexküll 1928, p. 162, see above). That the leading vitalist regarded Spemann, the “discoverer of an organizer,” as advocating against a purely materialist approach in biology becomes clear from their letters. In 1932, Uexküll wrote to Spemann:

But what you have found leads much further. You attack the problem of the active natural factors themselves. I am very curious about your article. It is easier for a camel to go through the eye of a needle than for a mechanistic explanation to go through your approach. However, my experience suggests the camels get through everywhere.^63^

Uexküll (1929) compared Spemann’s “organizer” with an “*Erbauungsbauplan*” [construction plan] or with his idea of a “mechanisator,” which for Uexküll was first and foremost an “active *Bauplan*” that he, in his fight against a materialist reductionism and mechanist thinking in biology, applied to overwrite mechanistic terminology with a more holistic one. For Uexküll, Spemann’s work on the *organizer* showed exactly what his idea of a system approach to “*Funktionskreise*” [functional circuits] in the living world had revealed, namely, that active *plans* could interact and that these patterns and systems had to be explored for a real understanding of the living world. A narrow analysis of just the material conditions, on the other hand, would remain unsatisfactory. Other scientists also put the idea of *Planmäßigkeit* and notions of a *Gestaltungsvermögen* [capacity for formation, creative ability] at the center of their interpretation of the essence of the *organizer,* as Walther Vogt’s contribution to the *Festschrift* for Spemann’s sixtieth birthday in 1929 shows. Vogt surely belonged to those scientists outside of the “Spemann school” (Fäßler 1996) whose embryological research on amphibia developed along similar lines to the work in Freiburg. It was also Vogt who prefaced the entire *Festschrift* with a quotation from Goethe’s writings on morphology (1817): “*Und keine Zeit und keine Macht zerstückelt / Geprägte Form, die lebend sich entwickelt*” (Vogt 1929).^64^

The debates on holism in Germany in the first decades of the twentieth century were rooted in an assemblage of different discourses and elements, whereby references such as Vogt’s to Goethe and to natural philosophy of the Romantic period often had a high symbolic value. Besides the symbolic value, Goethe’s morphology had, as recent studies show, a strong aftermath in various theory developments in early twentieth century (Axer et al. 2021). Theoretically, however, even more important for approaches such as Driesch and von Uexküll’s was a specific re-interpretation of Immanuel Kant. This influence is not surprising because Neo-Kantianism had been widespread and extremely influential in German philosophy since the late nineteenth century. As Anne Harrington and others have shown, a psychological interpretation of Kant was fundamental to Uexküll’s holistic approach (Harrington 1999, pp. 44–48; Esposito 2020). Driesch himself explained in his book *Der Vitalismus als Geschichte und Lehre* [The History and Theory of Vitalism] that his engagement with Kant’s *Critique of Judgement* was for him one of the most important issues in this book (Driesch 1905, p. vii). Thus, a popular version of Kant was also in the air for biologists at that time. This is not surprising because in the vitalist and also in the holistic debates, the problem of purposiveness in nature [*Zweckmäßigkeit*] was a central interest: specifically, whether this purposiveness could be explained by reducing it to the outcome of completely material and mechanistic conditions or whether it was an emergent property or even indicated a lawfulness peculiar to nature.

Kant’s approach to teleology was an essential point of reference in these debates. Given this discursive background in the early twentieth century, a term like the *organizer*, with meanings related to the living being as an organized system, also touched on (popular) philosophical issues that were ultimately rooted for many contemporaries in the philosophical horizon of problems that could be traced back to Kant’s view of the organism and nature in his *Critique of Judgement* (1790). Spemann himself did not refer explicitly to Kant in his writings, but Fäßler notes that Spemann participated in an informal philosophical discussion group at the university of Freiburg in the late 1920s that was initiated by Jonas Cohn, a Neo-Kantian (and former assistant of Edmund Husserl) who had strong interests in experimental psychology. This group, the *Pentathlon*, which included Friedrich Oehlkers (botany), Gustav Wolf (psychiatry), Gustav Mie (mathematics), Engelbert Krebs (theology), Spemann, and Cohn, met monthly to discuss interdisciplinary themes from a philosophical point of view (Fäßler 1997, pp. 96–97). Although Spemann himself never made an explicit statement that the notion of an *organizer* was in any way related to these philosophical discourses, his colleague and friend Fritz Baltzer suggested early on that the underlying idea of purposiveness in the concept of an *organizer* in Spemann’s work could also be seen as akin to the Kantian notion of teleology (Baltzer 1942, pp. 235–236).^65^

Baltzer himself, however, favored a materialist approach and seemed to be highly skeptical of any—even rhetorical—references to Goethe or German Romantic *Naturphilosophie.* When biochemical experiments on the *organizer* started to flourish around 1930, Baltzer wrote in a letter to Spemann:

How do you feel about the materialized organizer? What you wrote sounds good. I am always again convinced that we derive a deeper layer of knowledge, if such *Homunculus* experiments turned out to be successful. Vogt (who has accepted a call to Zürich) has a contrary opinion. For him, the organizer is an indivisible living whole (“und keine Macht und keine Kraft zerstückelt – geprägte Form,” etc. [a reference to Goethe, C.B.]). However, I believe that one has to break it into parts, morphological as well as physiological-material. I believe that animal physiology has already advanced largely along this way.^66^

## After the Nobel Prize: Leadership Analogies

Finally, a few remarks on the political dimension of the manifold meanings of the *organizer* are needed. It is important to note that Horder and Weindling (1986) indicated a political dimension to the *organizer*, but, as argued above, Spemann certainly did not introduce this notion with a hierarchical sense in mind. There were some lines of reception of Spemann’s work that emphasized an analogy to the political realm (for example, the zoologist Wilhelm Goetsch, who metaphorically compared Spemann’s *organizer* with a political organizer in 1926 [Goetsch 1926, p.1016]).^67^ However, in subsequent years, and especially after 1933 when the Nazis came to power, the understanding of the *organizer* as a clear hierarchical authority gained ground, particularly in the public reception of Spemann’s work. With the rise of the National Socialism in Germany, Spemann’s work took on a clear political connotation and was received by the public as supporting hierarchical ideas of “leadership” and control, and not just in Germany.

The award of the Nobel Prize to Spemann in 1935 was probably a crucial moment for this shift, because this event suddenly turned Spemann into a public figure for a broad non-scientific audience. A closer look at some of the newspaper and journal articles from that time is very illuminating. A report in *Science* on the 300th anniversary of Harvard University, which awarded Spemann an honorary doctorate in 1936, began as follows: “Chemical commanders in the bodies of embryo animals, giving orders that are received and obeyed by the developing parts, were described by Prof. Hans Spemann of the University of Freiburg, Nobel prizewinner.”^68^ On the occasion of the Nobel ceremony in December 1935, a Swedish newspaper ran the headline: “Organisms’ *Führer* works against cancer.”^69^ Spemann’s own behavior at the Nobel Prize ceremony seemed to have fostered the rhetorical analogy between Spemann’s person and the dictatorial system in Germany. A few German newspapers reported that Spemann had received the prize documents by performing the “German salute” (that is, the so-called *Hitlergruß*).^70^ From the archival material one cannot reconstruct whether Spemann truly behaved in that way in Stockholm or how far these reports themselves were part of Nazi propaganda. However, Spemann’s speech at the banquet is documented, and he clearly evinced a nationalist attitude, specifically by referring to himself as a son of the German nation whose main wish was to coexist peacefully and on a par with other nations.^71^

Almost every German newspaper reported on the Nobel prizewinner and described Spemann’s work as a kind of paradigm shift in the life sciences. Here, too, the press often highlighted a hierarchical and authoritarian dimension to the *organizer*, as scientists sometimes also did themselves. Otto Mangold wrote in March 1936 that the “organizer” was responsible for “determining the fate” [*Schicksalsbestimmung*] of the parts of the embryo, a statement with clear political undertones.^72^ The Swiss *Neue Zürcher Zeitung* (NZZ) announced the “mastery of animal development,” and the *Kölnische Illustrierte Zeitung* reported that Spemann’s “organizer” was of a higher order than the heredity factors, because the *organizer* was a “*Ganzheitsfaktor*” [holistic factor] to which all the other parts had to subordinate themselves.^73^

Besides such clearly political metaphors, Spemann’s vitalism also found a specific and pointed echo in public reports. *Der Alemanne* reported on Spemann’s speech at a Nobel ceremony organized by the city of Freiburg. The local newspaper summarized Spemann’s talk, and the journalist emphasized that Spemann had vanquished scientific materialism by demonstrating that “ultimately everything is spirit.”^74^ Another newspaper described Spemann’s revolutionary discovery as a “miracle of development” and referred to its fundamental importance for the *Weltanschauung*: Spemann’s work had shown that the “laws of development of a living being can never be compared to the laws of the world of the nonliving.”^75^ Finally, the *Frankfurter Zeitung*, one of the main German newspapers printed in the city where Goethe was born, celebrated Spemann’s research as a kind of victory of the Goethean approach over mechanism.^76^

## Conclusion

What can be concluded from the historical analysis of Spemann’s research style and the metaphorical dynamics related to the vague concept of an *organizer* in the interwar period? How can Spemann’s holism be understood historically? In this article, I have tried to show that his holistic approach clearly had vitalistic overtones, although one must be careful not to equate this with a naïve kind of spiritualism or even mysticism. Spemann’s holistic approach aimed at understanding the organism as a self-regulating system, and it rejected any kind of comparison of the living being with machine-like entities. Precisely here, Spemann’s metaphors and analogies that compared the realm of the living with psychic processes—or agency in nature with the behavior of an artist—should be understood in the broader cultural context of that time, in which holistic thinking and the emphasis on the creative ability of processes of formation were widespread, not only in scientific discourses. Against this broader background, it becomes clear that Spemann was often seen by his German contemporaries as being a representative of a modernist way of doing biology because of his clear rejection of late nineteenth-century mechanistic approaches and related orientations toward a narrow idea of causal analysis. When Spemann himself emphasized that he had frequently used terms that “designate psychological and not physical analogies” and that these analogies “were not meant to be just poetic analogies” (Spemann 1936, p. 278), these statements should be interpreted within this broader context: as statements in which a tentative search for an autonomous epistemic status of biology became articulated.

On a discursive level, such statements were in line with other approaches that located biology between the natural sciences and *Geisteswissenschaften* [humanities] at that time. Spemann’s holism (with its vitalistic connotation) became particularly expressed in the vague notion of an “organizer” and the metaphorical descriptions that can be found in Spemann’s texts. The intermingling of different discursive elements—the combination of modernity with traces of the past (such as the amalgamation of a modern experimental approach with self-descriptions of handicraft and former artistic practices)—was characteristic of the approach of Spemann and his group. The concept of an *organizer* also contained both a modern perspective on biological processes in terms of systems and functions and an anthropomorphic connotation that hinted at an organizing agency in the realm of living beings.

I have tried to show that the multiple layers of different meanings of the concept—its biological, philosophical, as well as political connotation—provided a productive tension. From an epistemological perspective, these ambiguities and tensions allowed a widespread reception of the concept by very different scientific approaches in the 1920s and 1930s, which then became a central element in debates about holism, vitalism, and reductionism at the time.

## Footnotes

1 These approaches are sometimes simplistically framed under the label of a *Lebensphilosophie* that distanced itself from a philosophical tradition of rationality. Paul Forman’s (1971) classical work on Weimar culture and quantum physics argued for a linkage between the intellectual atmosphere in Weimar Germany, critics of deterministic and materialist approaches, and the origins of quantum physics. Forman’s thesis encouraged numerous approaches in the history of science to understand scientific developments (beyond physics) in Germany in the interwar period against this background of a cultural atmosphere of crisis, with the intermingling of *Lebensphilosophie* and an anti-modernist, hostile intellectual milieu that cultivated a feeling of a crisis of science. However, to view *Lebensphilosophie* only as an irrational and anti-modernist movement is historically and philosophically too simplistic. For a more precise philosophical and historical discussion of various approaches of *Lebensphilosophie* (Nietzsche, Dilthey, Bergson, Simmel, and Plessner), see, for example, Hartung (2012). For a discussion of the reception of the Forman thesis in the history of science, see Trischler et al. (2008).

2 On the institutional struggles in the field of (evolutionary) morphology and the disciplinary split between comparative anatomy and embryology at German universities in the second half of the nineteenth century, see Nyhart (1987, 1995).

3 For a general history of models, theories, and approaches in experimental embryology in the late nineteenth and early twentieth century, see Horder et al. (1986), Maienschein (1991a, b, 1997), Gilbert (1991), Mocek (1998), Haraway [1976] 2004), Laubichler and Maienschein (2007).

4 “Eine idealistische Zeit sagte: Die Idee des Ganzen ist in jedem Teile enthalten. Aber auch in unserer realistischen Periode kann man nicht darum herum, die Summe der Möglichkeiten, welche zur Bildung des Ganzen gehören, auch im Teil vorhanden zu denken. … Driesch glaubte sich gezwungen, sich dieses das Ganze hervorbringende Etwas unräumlich zu denken, als eine intensive Mannigfaltigkeit, zu deren Bezeichnung er das alte Wort *Entelechie* wieder einführte. Es ist im Grunde nichts so sehr viel anderes als was mit der (auch unräumlichen) Idee gemeint war. Auch wenn man nicht glaubt, mit Driesch diese letzte Folgerung ziehen zu müssen, bleibt das äquipotente System, welches der harmonischen Gliederung fähig ist, ein reales Problem; ein Problem, welches auch dadurch nicht aus der Welt geschafft wird, daß die ‚harmonisch-äquipotentiellen ‘ Systeme beides vielleicht nie ganz in dem Sinne sind, in welchem Driesch es meinte.” (Spemann 1936, p. 224). An English translation of the book was published 1938 with a slightly different title: *Embryonic Development and Induction* (Spemann 1938).

5 „Mit diesen Tatsachen scheint mir in das bisher unangreifbare Gefüge des harmonisch-äquipotentiellen Systems eine Bresche gelegt, durch welche die experimentelle Analyse eindringen kann.” Zur Theorie der tierischen Entwicklung. Rektoratsrede von Prof. Ord. Hans Spemann, Geh. Reg.-Rat, Freiburg 1923, p.15, University Archive Freiburg, Spemann Papers, C 181, Signatur 75.

6 In 1907, Spemann explained to Roux that the term *Entwicklungsphysiologie* would be a “more sympathetic” name for the field [“sympathischere Bezeichnung”]. Spemann to Roux, 15. II. 1907, Universitätsbibliothek J.C. Senckenberg, Frankfurt, Spemann Papers, Na 47, Folder 4.

7 On August Weismann, see Churchill (2015); on Spemann’s discussion of Weismann’s theories in the 1920s, see Brandt, “Development and Heredity in the Interwar Period: Hans Spemann and Fritz Baltzer on Organizers and Merogones”, in this issue.

8 Hans Spemann, “Der Forscher,” Vortrag aus der Vortragsreihe der Volkshochschule Freiburg Der Mensch im Dienst der Gesellschaft,” 1932, unpublished manuscript, University Archive Freiburg, Spemann Papers, C 181, Signatur 115.

9 “die Dinge einzeln in ihren ewigen Verhältnissen zu schauen,” Hans Spemann, “Der Forscher,” p. 14, unpublished manuscript, University Archive Freiburg, Spemann Papers, C 181, Signatur 115.

10 “Aber es ist in der Tat ein kolossales Vergnügen, wenn man ein lebendes Objekt zwingen kann, etwas zu tun, was es ohne des verehrten Herrn Experimentators Willen nicht getan hätte.” Hans Spemann, “Der Forscher,” pp. 13–14, unpublished manuscript, University Archive Freiburg, Spemann Papers, C 181, Signatur 115.

11 “Ein anderer Typ, der in der Gegenwart besonders hoch geschätzt ist, ist der Experimentator, der Techniker im weitesten Sinne. Er sieht die Dinge nicht nur in ihrer Fülle vor sich ausgebreitet und wünscht sie in ihrer natürlichen Ordnung zu sehen, sondern empfindet sie als lebendige strömende krafterfüllte Triebe. In diesen Zusammenhang einzudringen, sich selbst in diesen Zusammenhang hineinzustellen, ist sein Ziel. Nicht nur, weil er glaubt, daß auf diesem Wege tiefe Einsicht zu gewinnen ist.” Hans Spemann, “Der Forscher,” p. 13, unpublished manuscript, University Archive Freiburg, Spemann Papers, C 181, Signatur 115.

12 “Wenn der experimentierende Forscher den tiefen inneren Drang hat, in das dynamische Getriebe einzudringen, sich selbst als mitschaffend hineinzustellen, wenn er dann sein eigenes Lebendigsein am stärksten fühlt, so hat dieser Typ [das ist der “ruhige, uninteressierte Schauer,” C.B.] das Bedürfnis, die Welt ausgebreitet vor sich ruhen zu sehen, die Dinge einzeln in ihren ewigen Verhältnissen zu schauen.” Hans Spemann, “Der Forscher,” p.14, unpublished manuscript, University Archive Freiburg, Spemann Papers, C 181, Signatur 115.

13 “Schilderung der Methode und der Atmosphäre im Institut in Freiburg,” unpublished typescript, no author, no date, University Archive Freiburg, Spemann papers, C 0181, Signatur111. The document is a speech for Spemann’s 60^th^ birthday; from the content of the paper, it is probable that Fritz Baltzer was the author. Quotation from p. 4: “Waren es in früheren Malerschulen Farbenmischungen und Lasuren, so ist es hier die Glasnadel, die Haarschlingentechnik, die sorgfältigste Vorbereitung der Experimente, die eine rationelle Bearbeitung des Tritonkeimes ermöglichen.”

14 “Wissenschaftliche Erziehung und ‘handwerkliche’ Ausbildung gingen im persönlichen Gespräch von Mensch zu Mensch, von Hand zu Hand. Er war der ‘tätige Meister,’ dem die anderen es gleich zu tun bestrebt waren” (Rotmann 1943, p. 260).

15 See Spemann’s notebooks from 1897 to 1938, University Archive Freiburg, Spemann papers, C 181, Signaturen 42–65.

16 See, for example, Sabine Becker (2018) on the “*Primat des Visuellen*” [primacy of the visual] in the popular culture of the Weimar Republic. The years after 1918 brought about a widespread proliferation of visual media in several fields, ranging from everyday life, arts, and through journalism to political discourses, driven by the increasing ubiquity of photography and film. On visualization in the history of life sciences more generally, see Anderson and Dietrich (2012); on visualization and standardization in the history of embryology, see Hopwood (2005).

17 Each picture card should consist of the image, a short description, the name of the scientist, and the date of the first publication of the result. The *Bildarchiv* began in 1920 with a collection of photographs from embryological experiments that were presented to Wilhelm Roux on the occasion of his 70^th^ birthday. However, the *Bildarchiv* was not successful; an outstanding historical desideratum is to analyze why this venture failed.

18 “Bauen Zeichnung und Wandtafel die festen Grundvorstellungen auf, so helfen Photographie und Projektion sie lebendig zu machen, eben dadurch, daß sie dem Bilde die zufälligen, individuellen Züge wiedergeben, durch deren Unterdrückung die eindringliche Klarheit der Zeichnung erreicht worden war. Dies gilt, soviel ich sehe, für jeden wissenschaftlichen Vortrag; im besonderen Maße aber da, wo das Bild nicht nur erklären, sondern in erster Linie auch beweisen soll. Je klarer und eindeutiger die Tatsache (z.B. die Folgen eines experimentellen Eingriffs) sind, welche irgend einen Satz beweisen, umso lebhafter wird der Wunsch sein, *sie in ihrer ganzen Unmittelbarkeit reden zu lassen*” [emphasis mine] (Spemann 1920, p. 303).

19 “Diese Entwicklung harmonisch-äquipotentieller Systeme mutet uns seltsam vertraut an. Wir haben eine Art von unmittelbarem Verständnis für dieses Störungen überwindende Festhalten am vorgesteckten Endziel. Wir werden an eigene Fähigkeiten erinnert, z.B. an unsere Fähigkeit, Tonintervalle richtig zu treffen und eine bekannte Melodie von jedem Grundton aus richtig zu singen. Die Natur verfährt bei der Entwicklung geradeso, wie der Künstler es tut, wenn er eine Zeichnung oder plastische Figur anlegt, ja wie jeder Organisator vorgeht, welcher über ein gegebenes Material, sei es belebt oder unbelebt, zu disponieren hat. Und im Grunde ist es auch nicht weiter verwunderlich, wenn ein Prinzip, nach welchem der ausgebildete Organismus funktioniert, sich schon bei seiner Entstehung wirksam zeigt.” Zur Theorie der tierischen Entwicklung. Rektoratsrede von Prof. Ord. Hans Spemann, Geh. Reg.-Rat, Freiburg 1923, p. 13, University Archive Freiburg, Spemann Papers, C 181, Signatur 75.

20 “Wenn es erlaubt ist, bei einem Anlaß wie dem heutigen für einen Augenblick die werktägliche Nüchternheit des exakten Forschers beiseite zu setzen, so will ich bekennen, daß ich bei meinem experimentellen Arbeiten oft das Gefühl einer Zwiesprache habe, bei der mir mein Gegenüber als der bedeutend Gescheitere vorkommt.” Zur Theorie der tierischen Entwicklung. Rektoratsrede von Prof. Ord. Hans Spemann, Geh. Reg.-Rat, Freiburg 1923, pp. 15–16, University Archive Freiburg, Spemann Papers, C 181, Signatur 75.

21 “Die Begegnung [mit Pauly, C.B.] war für mich von entscheidender Bedeutung: Es befestigte sich in mir die mir so gemäße und auch schon vorher angebahnte Grundüberzeugung, daß der Organismus in allen seinen lebendigen Teilen ‘beseelt’ ist; nicht weniger, wenn auch in anderer Ausprägung als das Organ, von welchem wir diese Funktion an uns selber kennen, das Gehirn. Von dieser grundsätzlichen Verwandtschaft aller Lebensvorgänge bin ich heute fester überzeugt denn je” (Spemann 1943, p. 167). Hamburger (1999) translates the word *beseelt* (which has a clear religious connotation in German) with “possesses an element of consciousness”: “It strengthened my natural conviction that all part of an organism possesses an element of consciousness – not just the organ with which we are familiar, the brain. Today, I am more convinced than ever before of the fundamental affinity of all living processes” (Hamburger 1999, p. 242).

22 For Schaxel’s and Bertalanffy’s critiques of Weismann and Roux’s “Maschinentheorie,” see Schaxel (1922, pp. 63–70); Bertalanffy (1928, pp. 109–125).

23 On *Tierpsychologie* [animal psychology], see Sucker (2002, pp. 31–34) and Gruevska (2022).

24 On Petersen, see Elze (1961).

25 Petersen (1937, p. 425): “Unser erster und am meisten kritischer experimentierender Embryolog stellt also gegen Ende seiner langen und überaus erfolgreichen Arbeit fest, daß man beim Studium eines so spezifisch lebendigen Geschehens … sich nicht irgendwelchen von außer- oder unterhalb des Lebendigen genommenen Erklärungsprinzipien nähert. Wenn er sich vielmehr veranlaßt sieht, schon zur Beschreibung und Verdeutlichung der Tatsachen seine Vorstellungen einem höheren Bereiche lebendigen Geschehens, dem des menschlichen Seelenlebens zu entnehmen, so dürfen wir das als einen Beweis dafür nehmen, daß die Biologie sich aus den Fesseln einer apriorischen mechanistisch-materialistischen Gedankenwelt zu befreien im Begriffe ist, und unabhängig und selbständig ihre Begriffe und Vorstellungsweisen ihrem eigenen Gebiet lebendigen Geschehens entnehmen will, also eine autonome Wissenschaft wird vom autonomen Leben”.

26 In contrast to that view, Harwood (1996, p. 366) has argued that “by all indications the dominant stance among German biologists during the Weimar Republic was *mechanistic materialism*.” However, while this assessment might be appropriate with respect to the emerging genetics community, given the broad field of biological approaches at that time, it is, in my view, doubtful that *mechanistic* materialism was the dominant discourse. Of course, most biologists were materialists, but my impression is that critics of a purely mechanistic approach were very widespread, and a lot of biologists had preferences for more complex non-reductionist models in biology.

27 On Alfred Kühn, see Rheinberger (2000), Harwood (1993, pp. 229–245).

28 Alfred Kühn, Hans Spemann, Sonderdruck aus Jahrbuch der Akademie der Wissenschaften zu Göttingen, 1941/1942, p. 102, University Archive Freiburg, Spemann papers, C 181, Signatur: 107; “Das Experiment war ihm nicht ein Abzwingen mit Hebeln und mit Schrauben, sondern wie eine Zwiesprache mit einem lebendigen ‘Arbeitspartner.’” The phrase: “Abzwingen mit Hebeln und mit Schrauben” (“forcing with levers and screws”) is a quotation from Goethe´s *Faust* I, from the scene of Faust’s monologue at the beginning: “Geheimnisvoll am lichten Tag/ läßt sich Natur des Schleyers nicht berauben, und was sie deinem Geist nicht offenbaren mag, das zwingst du ihr nicht ab mit Hebeln und mit Schrauben”.

29 Kühn to Spemann, 9 July 1929, quoted in Harwood (1993, p. 244).

30 Vogt (1929, p. xv): “Intuition, Phantasie, Einfühlung in das Wesen der Gestaltung hat Ihre Begriffe und neue Methoden hervorgebracht und so die großen Linien vorgezeichnet, den Plan in das Ungeordnete weil Unbekannte hinein schrittweise entworfen.”

31 “Innerhalb des Ganzen der Wissenschaft, die wir Naturwissenschaft nennen, sucht sich heute die Biologie gegen die Tyrannei der Physik und Chemie zu schützen.” (Heidegger [1929/1930] 1983, pp. 277–278).

32 „Führerschaft eines Forschers besteht nicht im Überraschenden und Ungewöhnlichen seiner Entdeckungen, sondern in der Ursprünglichkeit seines Verwachsenseins mit dem elementarsten Sachgehalt seines Gebietes “ (Heidegger 1983 [1929/1930], p. 279). Heidegger’s metaphor of a “*Verwachsenheit*” not only points to an intimate relationship, but the meaning of “growing together” also points to a botanical notion of grafting. Interestingly enough, in the German texts Spemann’s embryological transplantation experiments were still often described as experiments of “grafting” in the 1920s.

33 Letter from Heidegger to Spemann, 24 February 1929, quoted from Farias (1989, p. 141).

34 Letter from Spemann to his son Ulrich, Freiburg, 12 December 1939, “Der Autor [Weinstock, C.B.] scheint mir nämlich von Heidegger beeinflusst zu sein, und du weisst, dass der mir immer große Schwierigkeiten gemacht hat. Er hat eine mir so fremde Art zu denken, und von seinem Helden Hölderlin […] hat er vor allem die Dunkelheit übernommen, ohne dass er mir tief erscheint wie der ‘dunkle’ Heraklit.“ University Archive Freiburg, Spemann papers, C 181, Signatur 6.

35 On Uexküll’s scientific approaches, biopolitical and anti-Semitic writings, and his relation to National Socialist ideology, see Harrington (1999), Cheung (2004), Heredia (2020), Köchy (2020), Schnödl and Sprenger (2021).

36 “Jedem Musiker ist bekannt, daß man eine Melodie transponieren, d.h. aus anderen Tönen aufbauen kann, wenn bestimmte Beziehungen gewahr bleiben, die hier die Essentia ausmachen. Dementsprechend zerfällt die Musiklehre in eine Lehre von den Tönen und in eine Gestaltungslehre oder Kompositionslehre. Nach den gleichen Gesichtspunkten werden wir die Ontologie [sic] in eine Vererbungslehre im engeren Sinne, die sich mit den vertauschbaren Erbfaktoren befaßt, und in eine Lehre von den Bauprinzipien teilen, die ebenfalls eine Gestaltungslehre oder Kompositionslehre genannt zu werden verdient. Die erste Forschungsrichtung nennen wir nach ihrem Begründer ‘Mendelismus,’ die zweite sollte man nach dem Forscher, der hier die grundlegenden Prinzipien zutage gefördert hat, ‘Spemannismus’ nennen” (Uexküll 1928, p. 162).

37 “Wie es zweifellos richtig ist, daß jede geschriebene Note materiell ein Tintenklex ist, so ist zweifellos jede Eigenschaft der Lebewesen etwas materiell Festgelegtes. In den Eigenschaften der Lebewesen aber nichts anderes wahrnehmen zu wollen als den Ausdruck irgendeines Atomgezappels, ist nicht Schwerhörigkeit, sondern prinzipielle Taubheit. Bei dieser Sachlage ist es völlig aussichtslos, die Forscher, die nur mechanische und chemische Probleme sehen, davon zu überzeugen, daß es auch noch biologische Probleme gibt” (Uexküll 1928, p. 167).

38 The blastopore lip is the emerging structure in the developing embryo where the opening and infolding of tissue starts when the blastula stage turns into the gastrula stage through an invagination.

39 Spemann and Mangold (1924), Fäßler (1997, pp. 262–265) gives a detailed historical analysis of the developments of these experiments.

40 On the analysis of the work on the *organizer* and the role of the concept for twentieth-century biology, see Horder and Weindling (1986), Lenhoff (1991), Gilbert and Saxén (1993), Fäßler (1997), DeRobertis and Arechaga, eds. (2001), Sander and Fäßler (2001), Gilbert (2001), DeRobertis (2009).

41 This was a movement of Protestants and parts of the evangelical church against the control through Nazi organisations.

42 On boundary concepts, see Löwy (1992); on the dynamics of vague concepts in experimental systems, see Rheinberger (1997); on the role of metaphors in science, see, for example, Brandt (2004).

43 “Magic word” is a quotation from Hamburger (1969, p. 1123).

44 “So möchte ich hier betonen, daß ich meines Wissens nie eine Organisatortheorie aufgestellt habe. Ich habe das Wort Organisator geprägt, um gewisse neue, sehr merkwürdige Erscheinungen zu bezeichnen, welche mir bei meinen Experimenten entgegengetreten waren. Aber ich habe den Begriff von Anfang an als vorläufig betrachtet und wiederholt und ausdrücklich als vorläufig bezeichnet” (Spemann 1936, p. 275).

45 “Zu Beginn der Gastrulation wird die Individualität des Keims, könnte man sagen, repräsentiert durch die Zellen der oberen Urmundlippe, welche ein Organisationszentrum darstellen*,* von dem aus die übrigen wichtigen Teile des Körpers gebildet werden, und zwar wenigstens die Medullarplatte durch Angliederung von Zellen welche ein relativ noch indifferentes Material sind” (Spemann 1919, p. 591).

46 “Es wird also indifferentes Gewebe vom Organisator zu selbst organisierendem gemacht” (Spemann 1921, p. 569).

47 “Ein solches Stück eines Organisationszentrums kann man kurz einen ‚Organisator‘ nennen; er schafft sich in dem indifferenten Material, in dem er liegt oder in welches er künstlich verpflanzt wird, ein ‘Organisationsfeld’ von bestimmter Richtung und Ausdehnung.… Die oben mitgeteilten Ergebnisse der heteroplastischen Transplantation machen wahrscheinlich, daß der determinierende Einfluß ein vorwiegend auslösender ist, oder, um im Bilde zu bleiben, daß die organisatorische Tätigkeit, jedenfalls während der ersten Entwicklung, keine ‘instruierende,’ sondern eine ‘disponierende’ ist. Die nötige ‘Instruktion’ bringen die Zellen schon als Anlage mit” (Spemann 1921, pp. 568–569).

48 “Die Bezeichnung ‘Organisator’ (statt etwa ‘Determinator’) soll zum Ausdruck bringen, daß die von diesen bevorzugten Teilen ausgehende Wirkung nicht nur eine in bestimmter, beschränkter Richtung determinierende ist, sondern daß sie alle jene rätselhaften Eigentümlichkeiten besitzt, welche uns eben nur aus der belebten Natur bekannt sind” (Spemann and Mangold 1924, p. 637).

49 On Spemann’s discussion of Weismann’s theories in the 1920s, see Brandt (2022).

50 “Es soll heißen, daß diese Entwicklungsprozesse, wie alle vitalen Vorgänge, mögen sie sich einst in chemische und physikalische Vorgänge auflösen, sich aus ihnen aufbauen lassen oder nicht, in der Art ihrer Verknüpfung von allem uns Bekannten mit nichts so viel Ähnlichkeit haben wie mit denjenigen vitalen Vorgängen, von welchen wir die intimste Kenntnis besitzen, den psychischen. Es soll heißen, daß wir uns, ganz abgesehen von allen philosophischen Folgerungen, lediglich im Interesse des Fortschritts unserer konkreten, exakt zu begründenen Kenntnis diesen Vorteil unserer Stellung zwischen den Welten nicht sollten entgehen lassen” (Spemann 1936, p. 278).

51 When Spemann described reterospectively how he had conducted the transplantation experiments, he summarized his observations as follows: “Es war, wie wenn eine durch das Ìmplantat eingeführte organisatorische Kraft in dem von ihr beherrschten Bereich ohne Rücksicht auf Materialgrenzen geschaltet hätte.” A little later, he wrote: “Jene organisatorische Kraft endlich, welche über die Materialgrenzen weggreifend das Ganze gestaltet, stellt letzte Fragen des Entwicklungsgeschehens überhaupt zur Diskussion, für deren Inangriffnahme sich freilich kaum die ersten Möglichkeiten darzubieten scheinen” (Spemann 1936, pp. 94–95).

52 *DWDS. Der deutsche Wortschatz von 1600 bis heute* (https://www.dwds.de/wb/Organisator, accessed 15 November 2020): “Organisator: 1. Jemand der ein Vorhaben planmäßig vorbereitet, organisiert. 2. Jemand, der die Fähigkeit besitz, gut zu organisieren.”

53 DWDS; see, for example, the sources that are listed by the *Referenzkorpora* in DWDS on the usages of the word in the nineteenth century.

54 As the analysis of DWDS shows, the (quantitative) curve of the word usages increases exponentially from around 1900 to the late twentieth century, where there is more or less a high plateau of the usage of the word after 1950.

55 Huxley general correspondence, 1920–1924, Rice University, Woodson Research Center, Fondren Library, Huxley papers, box 6–8.

56 Huxley to Spemann, 26 September 1923, Universitätsbibliothek J.C. Senckenberg, Frankfurt, Spemann papers, Na 47, Nr. 3.

57 Spemann to Huxley, 5 January 1924, and 23. February 1924, Rice University, Woodson Research Center, Fondren Library, Huxley papers, box 8, folder 1–3.

58 Spemann to Huxley, Amsterdam, 31. 3. 1924, Rice University, Woodson Research Center, Fondren Library, Huxley papers, box 8, folder 1–3.

59 G. de Beer to Spemann, 5. February and 18. February 1926, Universitätsbibliothek J.C. Senckenberg, Frankfurt, Spemann papers, Na47, Nr.1.

60 G. de Beer to Spemann, 22 Oct 1927, Universitätsbibliothek J.C. Senckenberg, Frankfurt, Spemann papers, Na 47, Nr. 1.

61 On Schaxel, see Hopwood (1997), Reiß (2007), Baedke (2019).

62 Schaxel (1922, p. 69): “Mit solcher vorsichtigen Handhabung des Determinationsbegriffes wird die unzugängliche Starrheit der entwicklungsmechanischen Determinationsmaschine vermieden; aber es fragt sich, wie auf diesem Wege von den Erscheinungen zum Wesen der Dinge vorgedrungen werden kann, ohne daß Worte wie Zentrum und Streben zu weitgehenden theoretischen Folgerungen führen. An die Realisierung des Bildungstriebes der idealistischen Embryologen oder an epigenetische Konsequenzen wäre zu denken.”.

63 Uexküll to Spemann, 9 May 1932, Universitätsbibliothek J.C. Senckenberg, Frankfurt, Spemann papers, Na47, Nr. 5: “Aber was Sie gefunden haben, führt noch viel weiter. Sie rücken diese aktiven Naturfaktoren darselbst zu Leibe. Ich bin auf Ihren Aufsatz schon sehr gespannt. Eher geht ein Kamel durch ein Nadelöhr, als eine mechanische Erklärung durch Ihren Versuch. Aber nach meiner Erfahrung kommen die Kamele überall durch.”.

64 The quotation is from Goethe’s “Urworte. Orphisch” (1817) [Orphic Primal Words], “And neither time nor any power can dismember/Characteristic form, living, self-developing,” translation from Wetters (2014, p. 202).

65 “Wenn man aber fragt, ob Spemann Vitalist war, so wird man keine Antwort finden. Er hat sich selbst darüber nicht geäußert. Die Beweisführung Driesch schien ihm wohl nicht überzeugend. Viel eher mag ihm, dem exakten und kritischen Denker, eine Stelle in der teleologischen Urteilskraft Kants wegleitend gewesen sein. Zwar sei keine Hoffnung, sagt dort Kant, daß dereinst ein Newton auferstehen könne, der auch nur die `Erzeugung eines Grashalmes’ nach Naturgesetzen, die keine *Absicht* geordnet habe, begreiflich machen werde, aber es wäre „wiederum von uns zu vermessen geurteilt, die Möglichkeit absichtsloser Entstehung organisierter Wesen überhaupt zu leugnen” (Baltzer 1942, pp. 235–236).

66 Baltzer to Spemann, 9 Aug. 1930, Universitätsbibliothek J.C. Senckenberg, Frankfurt, Spemann papers, Na 47, Nr.1: “Wie ist es Dir mit … (den) in anderer Weise vermaterialisiertem Organisator gegangen? Was Du mir jetzt schriebst, klang gut. Ich bin [immer] wieder [überzeugt], dass man, wie solche Homunculus-Experimente gelingen, eine Schicht tiefer hin zur Erkenntniss kommt. Vogt (der einen Ruf nach Zürich angenommen hat) ist eher gegenteiliger Meinung. Für ihn ist der Organisator ein unteilbar lebendig Ganzes (und keine Macht und keine Kraft zerstückelt—geprägte Form usw.). Ich glaube aber doch, man wird es in Teile schlagen müssen, morphologisch, wie physiologisch-stofflich. Ich glaube, die Wirbeltierphysiologie ist schon ein großes Stück dieses Wegs gegangen….”

67 Goetsch (1926, p. 1011) was also aware about the political conditions for this analogy when he wrote: “Es ist wohl kein Zufall, daß in einer Zeitepoche, die überall neu organisierende Kräfte erstrebt, auch in der Biologie der Begriff des ‘Organisators’ aufgetaucht ist”.

68 “Harvard Conference Hears Scientists from Many Fields,” *The Science NewsLetter*, Vol. 30, No. 806 (September 19, 1936, p. 186).

69 Newspaper cutting with no name or date, “Organismens ‘Führer’ motverkar kräftsjukdom,” University Archive Freiburg, Spemann papers, C 181, Signatur 5.

70 Newspaper cutting with no name or date, handwritten annotation: Schwäbischer Merkur, 21. 12. 1935, “Der drohende Zeigefinger;” Braunschweigische Landes-Zeitung (no date): “Eine Abfuhr,” University Archive Freiburg, Spemann papers, C 181, Signatur 5.

71 “Ansprache beim Festbankatt” (sic), University Archive Freiburg, Spemann papers, C 181, Signatur 121.

72 “Zwillinge nach Wunsch! Schicksalsbestimmung der Keimteile, von Professor Dr. Otto Mangold,” no journal title, handwritten annotation: “Volk u. Welt,” Märzheft 1936, Verlag Volk und Welt, Herausg. Prof. Oppermann,” University Archive Freiburg, Spemann papers, C 181, Signatur 5. One should bear in mind that phrases such as “*Schicksalsbestimmung des deutschen Volkes*” were common in the political pathos of that time. These connotations are certainly not present in the English translation of “*Schicksalsbestimmung*” as the “determination of fate.”

73 Newspaper cutting with no name and date, handwritten annotation: NZZ (Neue Zürcher Zeitung), 13. 11. 35: “Die Beherrschung der tierischen Entwicklung,” Kölnische Illustrierte Zeitung (no date): “Die geniale Entdeckung,” p. 1559, University Archive Freiburg, Spemann papers, C 181, Signatur 5.

74 Der Alemanne (no date): “Freiburg ehrt seinen Nobelpreisträger,” University Archive Freiburg, Spemann papers, C 181 Signature 5; “… dass letzten Ende alles Geist ist.”

75 Newspaper cutting with no name and date: “Wunder der Entwicklung. Warum Spemann den Nobelpreis erhielt,” University Archive Freiburg, Spemann papers, C 181, Signatur 5; “… so bleibt doch die große weltanschauliche Bedeutung des Lebenswerkes Hans Spemanns unberührt: er hat uns gezeigt, daß die Gesetze der Entwicklung eines Lebewesens niemals mit den Gesetzen, die in der unbelebten Welt wirken, verglichen werden können.”

76 Newspaper cutting with no name and date, handwritten annotation: Frankfurter Ztg., 17.6. 1936: “Goethes Typusbegriff und die moderne Entwicklungsmechanik,” University Archive Freiburg, Spemann papers, C 181, Signatur 5.

## References

[CR1] Allen Garland E (2004). A Pact with the Embryo. Viktor Hamburger, Holistic and Mechanistic Philosophy in the Development of Neuroembryology. 1927–1955. Journal of the History of Biology.

[CR2] Allen Garland E (2005). Mechanism, Vitalism and Organicism in late Nineteenth and Twentieth-Century Biology: The Importance of Historical Context. Studies in History and Philosophy of Biological and Biomedical Sciences.

[CR3] Allen Garland E, Laubichler Manfred, Maienschein Jane (2007). A century of evo-devo: the dialectics of analysis and synthesis in twentieth-century life science. From Embryology to Evo-Devo. A History of Developmental Evolution.

[CR4] Allen Garland E, Maienschein Jane (1999). Introduction to Hans Spemann on Vitalism in Biology. Translation of a Portion of Spemann’s Autobiography, by Viktor Hamburger. Journal of the History of Biology.

[CR5] Anderson Nancy, Dietrich Michael R (2012). The Educated Eye. Visual Culture and Pedagogy in the Life Sciences.

[CR6] Ash Mitchell (1995). Gestalt Psychology in German Culture 1890–1967. Holism and the Quest for Objectivity.

[CR7] Axer, Eva, Eva Geulen, and Alexandra Heimes. 2021. *Aus dem Leben der Form. Studien zum Nachleben von Goethes Morphologie in der Theoriebildung des 20. Jahrhunderts*. Göttingen: Wallstein.

[CR8] Baedke Jan (2019). O Organism, Where Are thou? Old and New Challenges for Organism-Centered Biology. Journal of the History of Biology.

[CR9] Baltzer Fritz (1942). Zum Gedächtnis Hans Spemann. Die Naturwissenschaften.

[CR10] Bautzmann Hermann (1955). Die Problemlage des Spemannschen Organisators. Die Naturwissenschaften.

[CR11] Becker Sabine (2018). Experiment Weimar. Eine Kulturgeschichte Deutschlands 1918–1933.

[CR12] Bertalanffy Ludwig (1928). Kritische Theorie der Formbildung.

[CR13] Bertalanffy, Ludwig. 1930/1931. Tatsachen und Theorien der Formbildung als Weg zum Lebensproblem. *Erkenntnis* 1: 361–407.

[CR14] Brandt Christina (2004). Metapher und Experiment. Von der Virusforschung zum genetischen Code.

[CR500] Brandt, Christina. 2022. Development and Heredity in the Interwar Period: Hans Spemann and Fritz Baltzer on Organizers and Merogones. *Journal of the History of Biology*. 10.1007/s10739-022-09681-w 10.1007/s10739-022-09681-w PMC9468045 35930095

[CR15] Cheung Tobias (2004). From Protoplasm to Umwelt: Plans and the Technique of Nature in Jakob von Uexküll’s Theory of Organismic Order. Sign Systems Studies.

[CR16] Churchill Frederick B (2015). August Weismann. Development, Heredity, and Evolution.

[CR17] Conklin Edwin G (1936). Professor Hans Spemann. Nobel Laureate in Physiology and Medicine. The Scientific Monthly.

[CR18] DeRobertis Eddy M (2009). Spemann’s Organizer and the Self-Regulation of Embryonic Fields. Mechanisms of Development.

[CR19] De Robertis, Eddy M., and Juan Arechaga, eds. 2001. The Spemann-Mangold Organizer. Heritage of the 1924 Article by Hans Spemann and Hilde Mangold. Organizer Research Today. *The International Journal of Developmental Biology* 45: 1–378.

[CR20] Driesch Hans (1891). Entwicklungsmechanische Studien. I. Der Werth der beiden ersten Furchungszellen in der Echinodermenentwicklung. Experimentelle Erzeugung von Theil- und Doppelbildungen. Zeitschrift Für Wissenschaftliche Zoologie.

[CR21] Driesch Hans (1902). Über ein neues harmonisch-äquipotentielles System und über solche Systeme überhaupt. Archiv Für Entwicklungsmechanik Der Organismen.

[CR22] Driesch Hans (1905). Der Vitalismus als Geschichte und Lehre.

[CR23] Elze Curt (1961). Hans Petersen. Aus Anlaß der 15. Wiederkehr seines Todestages. Zeitschrift Für Anatomie Und Entwicklungsgeschichte.

[CR24] Esposito Maurizio, Michelini Francesca, Köchy Kristian (2020). Kantian ticks, Uexküllian Melodies, and the Transformation of the Transcendental Philosophy. Jakob von Uexküll and Philosophy.

[CR25] Farias Victor (1989). Heidegger und der Nationalsozialismus. Mit einem Vorwort v. Jürgen Habermas.

[CR26] Fäßler Peter E (1996). Hans Spemann (1869–1941) and the Freiburg School of Embryology. The International Journal of Developmental Biology.

[CR27] Fäßler, Peter E. 1997. *Hans Spemann 1896–1941. Experimentelle Forschung im Spannungsfeld von Empirie und Theorie. Ein Beitrag zur Geschichte der Entwicklungsphysiologie zu Beginn des 20. Jahrhunderts*. Berlin u.a.: Springer (Schriften der Mathematisch-naturwissenschaftlichen Klasse der Heidelberger Akademie der Wissenschaften, Nr. 1).

[CR28] Forman Paul (1971). Weimar Culture, Causality, and Quantum Theory, 1918–1927: Adaptation by German Physicists and Mathematicians to a Hostile Intellectual Environment. Historical Studies in the Physical Sciences.

[CR29] Gilbert Scott F (1991). A Conceptual History of Modern Embryology.

[CR30] Gilbert Scott F (2001). Continuity and Change: Paradigm Shifts in Neural Induction. The International Journal of Developmental Biology.

[CR31] Gilbert Scott F, Saxén Lauri (1993). Spemann´s Organizer: Models and Molecules. Mechanisms of Development.

[CR32] Goetsch Wilhelm (1926). “Organisatoren” bei regenerativen Prozessen. Die Naturwissenschaften.

[CR101] Gruevska, Julia. 2022. Analysis and/or interpretation in neurophysiology? A transatlantic discussion between F. J. J. Buytendijk and K. S. Lashley, 1929–1932.* Journal of the History of Biology*. 10.1007/s10739-022-09680-x 10.1007/s10739-022-09680-x PMC9467955 35678929

[CR33] Hamburger Viktor (1969). Hans Spemann and the Organizer Concept. Experientia.

[CR34] Hamburger Viktor (1984). Hilde Mangold, Co-Discoverer of the Organizer. Journal of the History of Biology.

[CR35] Hamburger Viktor (1988). The Heritage of Experimental Embryology. Hans Spemann and the Organizer.

[CR36] Hamburger Viktor (1999). Hans Spemann on Vitalism in Biology. Translation of a Portion of Spemann´s “Autobiography”. Journal of the History of Biology.

[CR37] Haraway, Donna Jeanne. 2004 [1976]. *Crystals, Fabrics, and Fields. Metaphors that Shape Embryo*s. Berkeley: North Atlantic Books.

[CR38] Harrington Anne (1999). Reenchanted Science: Holism in German Culture from Wilhelm II to Hitler.

[CR39] Harrison Ross (1933). Some Difficulties of the Determination Problem. The American Naturalist.

[CR40] Hartung Gerald, Schaede Stephan, Hartung Gerald, Kleffmann Tom (2012). Lebensphilosophie. Das Leben.

[CR41] Harwood Jonathan (1993). Styles of Scientific Thought. The German Genetics Community 1900–1933.

[CR42] Harwood Jonathan (1996). Weimar Culture and Biological Theory: A Study of Richard Woltereck (1877–1944). History of Science.

[CR43] Heidegger, Martin. 1983 [1929/1930]. *Die Grundbegriffe der Metaphysik*. *Welt- Endlichkeit- Einsamkeit* (Martin Heidegger, Gesamtausgabe, Bd. 29/30). Frankfurt a. Main: Vittorio Klostermann.

[CR44] Heredia Juan Manuel, Michelini Francesca, Köchy Kristian (2020). Jakob von Uexküll, an Intellectual History. Jakob von Uexküll and Philosophy.

[CR45] Hopwood Nick (1997). Biology Between University and Proletariat: The Making of a Red Professor. History of Science.

[CR46] Hopwood Nick (2005). Visual Standards and Disciplinary Change. Normal Plates, Tables and Stages in Embryology. History of Science.

[CR47] Hopwood Nick, Bowler Peter J, Pickstone John V (2009). Embryology. The Cambridge History of Science: The Modern Biological and Earth Sciences.

[CR48] Horder TJ, Witkowski JA, Wylie CC (1986). A History of Embryology.

[CR49] Horder TJ, Weindling PJ, Horder TJ, Witkowski JA, Wylie CC (1986). Hans Spemann and the Organiser. A History of Embryology.

[CR50] Huxley Julian (1912). The Individual in the Animal Kingdom.

[CR51] Huxley, Julian, and Gavin de Beer. 1963 [1934]. *The Elements of Experimental Embryology*. New York and London: Hafner Publishing Company.

[CR52] Köchy Kristian, Michelini Francesca, Köchy Kristian (2020). Uexküll’s Legacy: Biological Reception and Biophilosophical Impact. Jakob von Uexküll and Philosophy.

[CR53] Laubichler Manfred, Maienschein Jane (2007). From Embryology to Evo-Devo. A History of Developmental Evolution.

[CR54] Lenhoff Howard M (1991). Ethel Browne, Hans Spemann, and the Discovery of the Organizer Phenomenon. Biological Bulletin.

[CR55] Löwy Ilana (1992). The Strength of Loose Concepts: Boundary Concepts, Federative Experimental Strategies and Disciplinary Growth: The Case of Immunology. History of Science.

[CR56] Maienschein Jane, Gilbert Scott F (1991). The Origins of Entwicklungsmechanik. A Conceptual History of Modern Embryology.

[CR57] Maienschein Jane (1991). Epistemic Styles in German and American Embryology. Science in Context.

[CR58] Maienschein Jane (1997). Changing Conceptions of Organization and Induction. American Zoologist.

[CR59] Meyer Adolf (1929). Das Wesen der antiken Naturwissenschaft mit besonderer Berücksichtigung des Aristotelismus in der modernen Biologie. Sudhoffs Archiv Für Geschichte Der Medizin.

[CR60] Mocek Reinhard (1998). Die werdende Form. Eine Geschichte der kausalen Morphologie.

[CR61] Morgan Thomas Hunt (1927). Experimental Embryology.

[CR62] Nyhart Lynn (1987). The Disciplinary Breakdown of German Morphology, 1870–1900. Isis.

[CR63] Nyhart Lynn (1995). Biology Takes Form. Animal Morphology and the German Universities, 1800–1900.

[CR64] Oppenheimer Jane M (1970). Cells and Organizers. American Zoologist.

[CR65] Pauly August (1905). Darwinismus und Lamarckismus. Entwurf einer psychophysischen Teleologie.

[CR66] Petersen Hans (1937). Hans Spemann: Experimentelle Beiträge zu einer Theorie der Entwicklung. Zeitschrift Für Anatomie Und Entwicklungsgeschichte.

[CR67] Peterson Erik (2014). The Conquest of Vitalism or the Eclipse of Organicism? The 1930s Cambridge Organizer Project and the Social Network of Mid-Twentieth-Century Biology. British Journal of the History of Science.

[CR68] Peterson Erik (2016). The Life Organic. The Theoretical Biology Club and the Roots of Epigenetics.

[CR69] Reiß Christian (2007). No Evolution, No Heredity, Just Development—Julius Schaxel and the End of the Evo-Devo Agenda in Jena, 1906–1933: A Case Study. Theory in Biosciences.

[CR70] Rheinberger Hans-Jörg (1997). Toward a History of Epistemic Things. Synthesizing Proteins in the Test Tube.

[CR71] Rheinberger Hans-Jörg (2000). Ephestia: The Experimental Design of Alfred Kühn’s Physiological Developmental Genetics. Journal of the History of Biology.

[CR72] Rinard RG (1988). Neo-Lamarckism and Technique. Hans Spemann and the Development of Experimental Embryology. Journal of the History of Biology.

[CR73] Rotmann, Eckhard. 1943. Die Werkstatt. In *Hans Spemann*, *Forschung und Leben*, ed. Friedrich Wilhelm Spemann, 259–274. Stuttgart: J. Engelhorns Nachf. Adolf Spemann.

[CR74] Roux Wilhelm (1888). Beiträge zur Entwickelungsmechanik des Embryo V. Über die künstliche Hervorbringung halber Embryonen durch Zerstörung einer der beiden ersten Furchungszellen, sowie über die Nachentwicklung (Postgeneration) der fehlenden Körperhälfte. Virchows Archiv Für Pathologische Anatomie Und Physiologie Und Klinische Medizin.

[CR75] Sander Klaus, Fäßler Peter (2001). Introducing the Spemann-Mangold Organizer: Experiments and Insights that Generated a Key Concept in Developmental Biology. The International Journal of Developmental Biology.

[CR76] Schaxel Julius (1922). Grundzüge der Theorienbildung in der Biologie.

[CR77] Schnödl, Gottfried, and Florian Sprenger. 2021. *Uexkülls Umgebungen: Umweltlehre und rechtes Denken*. Lüneburg: Meson Press.

[CR78] Semon Richard (1904). Die Mneme als erhaltendes Prinzip im Wechsel des organischen Geschehens.

[CR79] Spemann Hans (1919). Experimentelle Forschungen zum Determinations- und Individualitätsproblem. Die Naturwissenschaften.

[CR80] Spemann Hans (1920). Ein Wissenschaftliches Bildarchiv. Roux Archiv Für Entwicklungsmechanik Der Organismen.

[CR81] Spemann Hans (1921). Die Erzeugung tierischer Chimären durch heteroplastische embryonale Transplantation zwischen Triton cristatus und taeniatus. Archiv Für Entwicklungsmechanik.

[CR82] Spemann Hans (1924). Vererbung und Entwicklungsmechanik. Die Naturwissenschaften.

[CR83] Spemann Hans (1924). Über Organisatoren in der tierischen Entwicklung. Die Naturwissenschaften.

[CR84] Spemann Hans (1927). Neue Arbeiten über Organisatoren in der tierischen Entwicklung. Die Naturwissenschaften.

[CR85] Spemann Hans (1927). Über Organisatoren in der tierischen Entwicklung. Forschungen und Fortschritt.

[CR86] Spemann Hans (1927). Croonian Lecture. Organizers in Animal Development. Proceedings Royal Society of London, B.

[CR87] Spemann Hans (1929). Organisatoren in der tierischen Entwicklung. Forschungen und Fortschritt.

[CR88] Spemann Hans (1936). Experimentelle Beiträge zu einer Theorie der Entwicklung.

[CR89] Spemann Hans (1938). Embryonic Development and Induction. Silliman Lectures.

[CR90] Spemann, Hans. 1943. *Forschung und Leben*, ed. F. Wilhelm Spemann. Stuttgart: J. Engelhorn Nachf. Adolf Spemann.

[CR91] Spemann Hans, Mangold Hilde (1924). Über die Induktion von Embryonalanlagen durch Implantation artfremder Organisatoren. Roux’s Archiv Für mikroskopische Anatomie Und Entwicklungsmechanik.

[CR92] Spemann Hans, Schotte Otto (1932). Über xenoplastische Transplantation als Mittel zur Analyse der embryonalen Induktion. Die Naturwissenschaften.

[CR93] Sucker Ulrich (2002). Das Kaiser-Wilhelm-Institut für Biologie. Seine Gründungsgeschichte, seine problemgeschichtlichen und wissenschaftstheoretischen Voraussetzungen (1911–1916).

[CR94] Trischler Helmuth, Carson Cathryn, Kojevnikov Alexei (2008). Beyond *Weimar Culture*—Die Bedeutung der Forman-These für eine Wissenschaftsgeschichte in kulturhistorischer Perspektive. Berichte Zur Wissenschaftsgeschichte.

[CR95] von Uexküll Jakob (1928). Theoretische Biologie. Zweite gänzlich neu bearbeitete.

[CR96] von Uexküll Jakob (1929). Plan und Induktion. Roux Archiv Für Entwicklungsmechanik Der Organismen.

[CR97] Vogt Walther (1929). Hans Spemann zum 60. Geburtstag. Roux Archiv Für Entwicklungsmechanik Der Organismen.

[CR98] Weismann August (1885). Die Continuität des Keimplasmas als Grundlage einer Theorie der Vererbung.

[CR99] Weismann August (1892). Aufsätze über Vererbung und verwandte biologische Fragen.

[CR100] Wetters Kirk, Wetters K (2014). Appendix: German Text and English Translation of Goethe’s “Urworte Orphisch” (with Commentary). Demonic History. From Goethe to the Present.

